# Exploring the Association Between Human Blood Metabolites and Autism Spectrum Disorder Risk: A Bidirectional Mendelian Randomization Study

**DOI:** 10.1002/hsr2.70528

**Published:** 2025-03-03

**Authors:** Wenhua Li, Suya Ma, Yunong Tian

**Affiliations:** ^1^ State Key Laboratory of Component‐Based Chinese Medicine, Ministry of Education, Key Laboratory of Pharmacology of Traditional Chinese Medicine Formulae, Institute of Traditional Chinese Medicine, Tianjin University of Traditional Chinese Medicine Tianjin China; ^2^ Guang'anmen Hospital, China Academy of Chinese Medicine Sciences Beijing China; ^3^ CangZhou Hospital of Integrated Traditional Chinese and Western Medicine in Hebei Province Hebei China

**Keywords:** autism spectrum disorder, blood metabolites, Mendelian randomization, network pharmacology

## Abstract

**Background and Aims:**

Autism spectrum disorder (ASD) is a complex neurodevelopmental condition with a poorly understood etiology. Recent studies have suggested that metabolic dysregulation might be linked to the development of ASD; however, causal relationships remain unclear. This study aimed to investigate the causal association between these factors using two‐sample Mendelian randomization (TSMR).

**Methods:**

We conducted a TSMR analysis to assess the relationship between blood metabolites and ASD using summarized GWAS data. The metabolite dataset from the Canadian Longitudinal Study of Aging included 1091 metabolites and 309 ratios from 7824 European individuals. The ASD data from the Psychiatric Genomics Consortium comprised 18,381 ASD cases and 27,969 controls. Blood metabolites were set as exposures with ASD as the outcome. We primarily used the inverse‐variance weighted method, supplemented by MR‐Egger, weighted median, simple mode, and weighted mode methods. We also conducted sensitivity analyses to confirm robustness. Replication, confounding, and reserve analyses were performed to verify causation. Additionally, metabolic pathway and network pharmacology analyses were conducted to explore potential mechanisms.

**Results:**

We identified 55 known metabolites including 13 metabolite ratios and 10 unknown blood metabolites associated with ASD. Additionally, our analysis identified 13 potential metabolic pathways, among which tryptophan metabolism was the most notable (*p* = 0.0388). Gene Ontology functional analysis and Kyoto Encyclopedia of Genes and Genomes analysis highlighted crucial pathways, such as cellular glucuronidation, glucuronosyltransferase activity, and bile secretion, and the significance of the apical part of the cell.

**Conclusions:**

Our findings indicate that the dodecenedioate, methionine sulfone, cysteine to alanine ratio and proline to glutamate ratio have an impact on ASD. These results enhance our understanding of the metabolic pathways involved in ASD and could lead to new avenues for intervention and prevention. Further research is needed to explore the mechanisms underlying these associations and confirm these findings in different populations.

## 1 Introduction

Autism spectrum disorder (ASD) is a complex neurodevelopmental condition characterized by challenges in social interaction, communication, and the presence of repetitive and restricted behaviors [1]. The etiology of ASD is not fully understood, with genetic and environmental factors and their interplay believed to play significant roles in its development [2, 3]. Recent research has focused on the role of blood metabolites in the pathophysiology of ASD [4, 5, 6, 7, 8]. Blood metabolites are small‐molecule compounds found in the blood, including lipids, amino acids, sugars, vitamins, and other metabolic byproducts [9]. These metabolites reflect the metabolic state of the body and may be implicated in the onset and progression of ASD [10]. Analyzing the blood metabolites of individuals with ASD may provide a better understanding of the biological processes involved in the disorder, potentially leading to new diagnostic and therapeutic insights.

Mendelian randomization (MR) is an epidemiological method that uses genetic variants as instrumental variables (IVs) to assess the causal relationship between exposure and disease outcomes [11]. The integration of network pharmacology with MR to analyze the impact of exposure on diseases allows the elucidation of complex biological networks and causal relationships between exposure and various health conditions [12]. MR helps establish causality and enhances the rigor and reliability of findings [13]. Network pharmacology maps these causal relationships onto broader biological pathways, providing a systemic view of the interactions and their effects on disease processes [12]. In summary, the analysis of blood metabolites in individuals with ASD, combined with MR and network pharmacology, offers a promising avenue for exploring the pathophysiological mechanisms of the disorder, with significant implications for future prevention, diagnosis, and treatment of ASD. Consequently, this innovative methodology has significant potential to advance precision medicine, offering more personalized and effective healthcare solutions based on individual metabolic and genetic profiles [14].

## 2 Materials and Methods

### 2.1 Study Design

**Figure 1:**
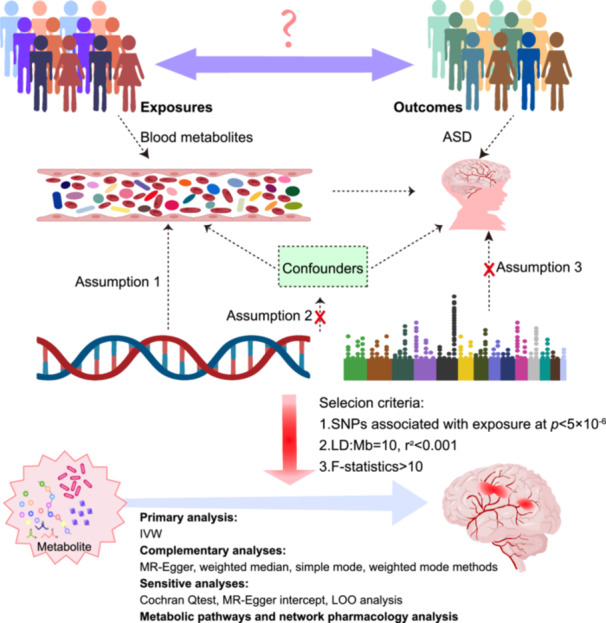
Illustration of the analytical framework of the study. Assumption 1: IVs are significantly associated with blood metabolites. Assumption 2: Asserts that IVs are independent of confounding variables. Assumption 3: The effect of IVs on ASD is mediated exclusively by blood metabolites. Abbreviations: ASD, autism spectrum disorder; IVW, inverse variance weighted; LD, linkage disequilibrium; LOO, leave‐one‐out; MR, Mendelian randomization; SNPs, single nucleotide polymorphisms.

This study employed a two‐sample Mendelian randomization (TSMR) approach to infer causality between genetic variants robustly associated with modifiable exposure and outcome. This TSMR method uses SNPs as IVs, which is highly reliable. There are three key assumptions that underlie MR [15]: Assumption 1: IVs are significantly associated with blood metabolites. Assumption 2: Asserts that IVs are independent of confounding variables. Assumption 3: The effect of IVs on ASD is mediated exclusively by blood metabolites (Figure 1).

### 2.2 Data Sources

Our primary exposure variable was the blood metabolite levels, and the outcome of interest was ASD. The data for ASD were sourced from the Psychiatric Genomics Consortium (PGC) (https://pgc.unc.edu/), which includes 18,381 ASD cases and 27,969 controls [16]. As the largest and most comprehensive resource in psychiatric genomics, the PGC dataset offers substantial statistical power to detect genetic associations with ASD. This dataset provides detailed information on genetic variants associated with ASD, which forms the basis for selecting IVs for our MR analysis. The blood metabolite data for this study were obtained from the largest genome‐wide association study (GWAS) data published in Nature Genetics in 2023 conducted by Chen et al [17]. This database encompasses 1400 meticulously quality‐controlled metabolites, including 1091 distinct metabolites, with 309 metabolite ratios [17]. We used the genetic associations of these metabolites to explore how genetic variations affecting metabolite levels may be related to ASD. Specifically, genetic variants that exhibited significant associations with metabolite levels were selected as IVs for MR analysis.

These metabolites were derived from a sample of 8299 participants in the Canadian Longitudinal Study on Aging (CLSA) cohort [17]. The CLSA dataset provides rich and well‐characterized metabolite data despite its focus on an aging population [18]. For the blood metabolite exposure variable, we emphasized consideration of the correlation with metabolites, independence, exclusivity, and biological plausibility. Therefore, this approach allows us to leverage the comprehensive metabolite profiles available in CLSA to explore broader biological mechanisms that may underlie ASD, independent of age‐specific effects [18, 19, 20].

### 2.3 Selection of Instrumental Variables

IVs were derived from 1400 blood metabolites and ASD, with genetic instruments chosen according to a stringent criterion of genome‐wide significance, specifically a *p*‐value threshold of less than 5 × 10^−6^. We established a linkage disequilibrium threshold of *r* ^2^ < 0.001 within a 10 Mb physical distance.

### 2.4 Mendelian Randomization Analysis

MR utilizes several statistical methods to estimate the causal effect of an exposure on an outcome, using genetic variants as IVs. Among these methods, IVW, MR‐Egger (ME), weighted median (WM), simple mode (SM), and weighted mode (W‐Mode) are commonly used [21], each addressing specific challenges and assumptions in the analysis. IVW is the most straightforward approach, in which each genetic variant's estimate of the exposure‐outcome effect is weighted by the inverse of its variance [22]. The IVW method assumes that all genetic variants are valid instruments [23]. It provides a precise estimate when all IVs are valid, but sensitive to pleiotropic effects [24]. MR‐Egger is particularly useful when some genetic variants are suspected to violate the exclusion restriction assumption, although this may lead to biased and inflated Type 1 error rates in practice [25]. However, the WM method reduces Type 1 errors, and enhances causal effect detection [26]. The W‐Mode method provides a robust estimate of causal effects by emphasizing the most frequently observed causal estimate across the largest valid SNP cluster, thereby reducing the influence of outliers and pleiotropic effects [27]. The SM method provides a robust alternative to IVW and is particularly effective against outliers and pleiotropic effects [28]. Therefore, the result of the IVW method is the most primary and important method [29], whereas the MR‐Egger, WM, SM, and W‐Mode methods were performed as additional tests to assess the robustness of the IVW results [30, 31]. In this study, we used the IVW approach as the main MR method to estimate the causal relationship between the metabolites and ASD. When the IVW method identified a causal association (*p* < 0.05), four additional MR methods (ME, WM, SM, and W‐Mode) were used to supplement the IVW results. All statistical analyses were performed using TSMR. Two‐tailed *p* < 0.05 was considered statistically significant. The odds ratios (OR) for all MR estimates are presented along with 95% confidence intervals (CIs) to measure the strength of association. An OR greater than 1 indicates an increase in the levels of the metabolite in individuals with ASD, whereas an OR less than 1 indicates a decrease.

### 2.5 Sensitivity Analysis

Sensitivity analyses are imperative in MR studies to ensure the reliability of MR estimates in the presence of potential bias [32]. To this end, a suite of methods, including Cochran's Q‐statistic, MR‐Egger, and LOO analysis [33], were employed to corroborate the stability of the significant findings (indicated by *p*IVW < 0.05). Among them, Cochrane's Q test was used to test heterogeneity among the IVs, with significant heterogeneity (*p* < 0 .05) [34]. Concurrently, we examined the presence of horizontal pleiotropy using the MR‐Egger intercept test [35]. In addition, the LOO analysis method evaluates the robustness of the overall estimate by sequentially excluding each genetic variant used as an instrument to ascertain that no single SNP affects the results [36, 37]. Furthermore, a funnel plot was used to detect asymmetry that could indicate potential pleiotropy or other biases among the genetic instruments used [38]. By conducting these sensitivity analyses, researchers can better understand the reliability of MR findings and the potential impact of bias on the causal inferences drawn from the study.

### 2.6 Replication Analysis

To further bolster the credibility and persuasiveness of our estimates, we performed an independent Instrumental Variable analysis with IVW utilizing ASD GWAS data sourced from the FinnGen project [39].

### 2.7 Confounding Analysis

To mitigate potential confounders in our MR analysis, we conducted a comprehensive sensitivity analysis using a battery of statistical tools to identify SNPs that may breach the MR assumptions. Although we aimed to minimize residual confounding factors, some SNPs persisted. To address this, we scrutinized the IVs for metabolite‐associated SNPs using the Phenoscanner V2 platform (http://www.phenoscanner.medschl.cam.ac.uk/). Our scrutiny process focused on checking for associations with established ASD risk factors, including gut microbiome (GM) [21], dietary factors [40], education and intelligence [41], body mass index (BMI) [42], and birth weight [43]. If we could detect SNPs tied to these known confounders, we would repeat the MR analysis, excluding these SNPs, to substantiate the stability of our findings.

### 2.8 Reserve Analysis

To investigate the influence of ASD on the composition of blood metabolites, a series of reverse MR studies were performed utilizing TSMR. During this analysis, stringent filtering of IVs was applied, with a significance threshold set at *p* < 5 × 10^−6^ and an *r* ^2^ criterion of less than 0.001 for any two IVs within a 10,000 kb region.

### 2.9 Metabolic Pathways and Network Pharmacology Analysis

To elucidate the biological mechanisms underlying the effects of blood metabolites in ASD. The chosen metabolite metabolic pathways were investigated using a web‐based platform MetaboAnalyst6.0 https://www.metaboanalyst.ca/. MetaboAnalyst6.0 platform can be used for comprehensive metabolomic data analysis, interpretation, and integration with other omics data. In this study, we focused our analysis exclusively on metabolites that surpassed the stipulated significance threshold, thereby qualifying for further assessment in metabolic pathway evaluation (*p*IVW < 0.05) [26]. Network pharmacology helps to understand the complex biological pathways involved in diseases, which can be crucial for identifying relevant genetic variants used in MR, and can help investigate reaction networks, key targets, and metabolites [44]. Related genes were derived from the MR and GEO databases (https://www.ncbi.nlm.nih.gov/geo/) [45]. Functional enrichment analysis was performed for the related genes. Enrichment analysis of Gene Ontology (GO) biological processes (BP), cellular components (CC), molecular functions (MF), and Kyoto Encyclopedia of Genes and Genomes (KEGG) pathways (http://www.bioinformatics.com.cn/).

## 3 Results

### 3.1 The Association Between Metabolites and ASD

After tightly controlling the quality of the IVs, 1400 metabolites were eventually captured in the MR study. All F‐values for SNP inclusion were greater than 10, suggesting the absence of potentially weak instrument bias.

**Figure 2:**
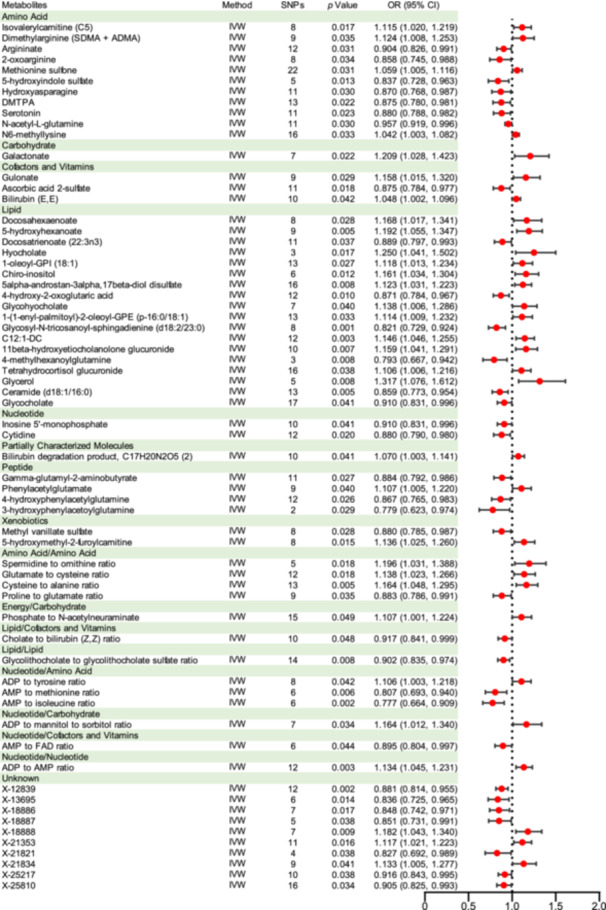
Forest plot for the causality of blood metabolites on ASD derived from IVW analysis. Abbreviations: ADP, adenosine 5′‐diphosphate; AMP adenosine 5′‐monophosphate; ASD, autism spectrum disorder; CI, confidence interval; DMTPA, 2,3‐dihydroxy‐5‐methylthio‐4‐pentenoate; FAD, Flavin adenine dinucleotide; IVW, inverse variance weighted; OR, odds ratio; SNPs, single nucleotide polymorphisms.

**Figure 3:**
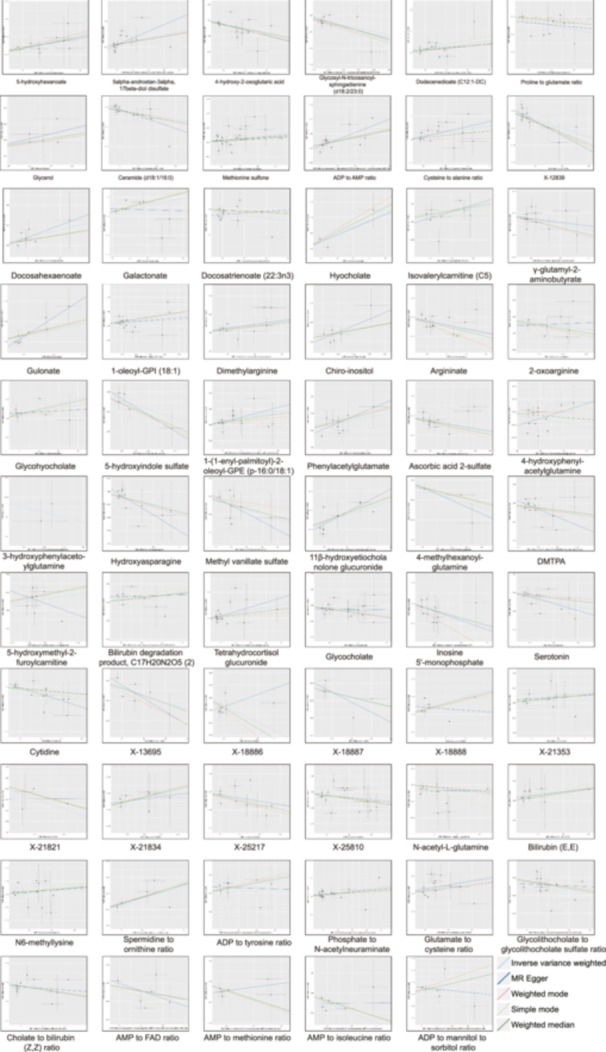
Scatter plots to visualize the causal associations between blood metabolites and ASD. The x‐axis shows the effect size of SNPs on metabolites levels, and the y‐axis shows the effect size of SNPs on ASD. Each point represents a different SNP. The different lines represent the results from models including different MR methods. Abbreviations: ADP, adenosine 5′‐diphosphate; AMP adenosine 5’‐monophosphate; ASD, autism spectrum disorder; DMTPA, 2,3‐dihydroxy‐5‐methylthio‐4‐pentenoate; FAD, Flavin adenine dinucleotide; SNPs, single‐nucleotide polymorphisms.

In the metabolic analysis associated with ASD, the IVW analysis initially identified 65 metabolites significantly linked to the disorder. These included 13 ratio indicators, 10 unidentified compounds, and 42 known metabolites (Figure 2). Among the identified compounds were sourced from eight metabolic groups, 11 from amino acids, 1 from carbohydrate, 3 from cofactors and vitamins, 18 from lipids, 2 from nucleotides, 1 from partially characterized molecule, 4 from peptides, and 2 from xenobiotics metabolism [17] (Table 1). Within the spectrum of known metabolites, 22 exhibited a positive correlation with ASD severity, whereas 20 showed a negative association. The metabolites identified in IVW analysis were further validated and corroborated using four complementary analytical methodologies. These supportive studies were aligned in parallel with the primary IVW outcomes (Figures S1–4, Figure 3, and Table 1). However, there were notable exceptions, including several distinct metabolites such as 2‐oxoarginine, 4‐hydroxyphenylacetylglutamine, docosatrienoate (22:3n3), 5‐hydroxymethyl‐2‐furoylcarnitine, galactonate, X‐18886, X‐18888, X‐21821, *N*‐acetyl‐l‐glutamine, adenosine 5’‐diphosphate (ADP) to tyrosine ratio, adenosine 5′‐monophosphate (AMP) to methionine ratio, AMP to isoleucine ratio, and ADP to mannitol to sorbitol ratio.

**Table 1:** MR estimates and sensitivity analyses of the causal relationship between blood and ASD.

Metabolites	Method	SNPs	OR		Heterogeneity		Pleiotropy		Increase/decrease risk
			*p* Value	OR (95% CI)	*Q*	*p* Value	Egger intercept	*p* Value	
Amino acid									
Isovalerylcarnitine (C5)	IVW	8	0.017	1.115 (1.020, 1.219)	7.151	0.413	−0.016	0.560	Increase
	ME	8	0.280	1.244 (0.868, 1.784)	6.725	0.347			
	WM	8	0.030	1.134 (1.013, 1.269)					
	SM	8	0.345	1.087 (0.925, 1.276)					
	W‐Mode	8	0.098	1.133 (0.997, 1.289)					
Dimethylarginine (SDMA + ADMA)	IVW	9	0.035	1.124 (1.008, 1.253)	3.891	0.867	0.003	0.864	Increase
	ME	9	0.457	1.102 (0.865, 1.405)	3.859	0.796			
	WM	9	0.233	1.094 (0.944, 1.267)					
	SM	9	0.492	1.086 (0.868, 1.358)					
	W‐Mode	9	0.407	1.086 (0.903, 1.305)					
Argininate	IVW	12	0.031	0.904 (0.826, 0.991)	5.517	0.904	0.001	0.959	Decrease
	ME	12	0.366	0.900 (0.723, 1.120)	5.514	0.854			
	WM	12	0.046	0.882 (0.779, 0.998)					
	SM	12	0.093	0.828 (0.677, 1.012)					
	W‐Mode	12	0.069	0.831 (0.695, 0.995)					
2‐Oxoarginine	IVW	8	0.034	0.858 (0.745, 0.988)	7.541	0.375	−0.023	0.431	Decrease
	ME	8	0.875	1.040 (0.651, 1.662)	6.740	0.346			
	WM	8	0.040	0.822 (0.681, 0.991)					
	SM	8	0.188	0.795 (0.584, 1.082)					
	W‐Mode	8	0.205	0.791 (0.570, 1.099)					
Methionine sulfone	IVW	22	0.031	1.059 (1.005, 1.116)	17.032	0.709	0.002	0.752	Increase
	ME	22	0.348	1.046 (0.954, 1.147)	16.929	0.658			
	WM	22	0.269	1.039 (0.971, 1.113)					
	SM	22	0.364	1.067 (0.930, 1.225)					
	W‐Mode	22	0.236	1.044 (0.974, 1.120)					
5‐Hydroxyindole sulfate	IVW	5	0.013	0.837 (0.728, 0.963)	2.178	0.703	0.019	0.453	Decrease
	ME	5	0.181	0.720 (0.496, 1.044)	1.439	0.696			
	WM	5	0.010	0.791 (0.662, 0.944)					
	SM	5	0.128	0.777 (0.600, 1.006)					
	W‐Mode	5	0.102	0.776 (0.613, 0.982)					
Hydroxyasparagine	IVW	11	0.030	0.870 (0.768, 0.987)	5.154	0.881	0.016	0.332	Decrease
	ME	11	0.115	0.736 (0.522, 1.038)	4.103	0.904			
	WM	11	0.110	0.878 (0.748, 1.030)					
	SM	11	0.305	0.859 (0.653, 1.131)					
	W‐Mode	11	0.261	0.859 (0.670, 1.103)					
2,3‐Dihydroxy‐5‐methylthio‐4‐pentenoate (DMTPA)	IVW	13	0.022	0.875 (0.780, 0.981)	13.340	0.345	0.027	0.245	Decrease
	ME	13	0.110	0.653 (0.404, 1.055)	11.732	0.384			
	WM	13	0.101	0.882 (0.758, 1.025)					
	SM	13	0.246	0.854 (0.662, 1.101)					
	W‐Mode	13	0.258	0.858 (0.666, 1.105)					
Serotonin	IVW	11	0.023	0.880 (0.788, 0.982)	5.348	0.867	0.008	0.654	Decrease
	ME	11	0.216	0.829 (0.629, 1.092)	5.134	0.823			
	WM	11	0.332	0.928 (0.799, 1.079)					
	SM	11	0.677	0.950 (0.752, 1.200)					
	W‐Mode	11	0.677	0.950 (0.752, 1.200)					
*N*‐acetyl‐l‐glutamine	IVW	11	0.030	0.957 (0.919, 0.996)	9.926	0.447	−0.019	0.057	Decrease
	ME	11	0.945	1.002 (0.946, 1.062)	5.167	0.820			
	WM	11	0.132	0.967 (0.926, 1.010)					
	SM	11	0.205	0.904 (0.781, 1.046)					
	W‐Mode	11	0.301	0.975 (0.931, 1.021)					
N6‐methyllysine	IVW	16	0.033	1.042 (1.003, 1.082)	13.593	0.557	0.006	0.515	Increase
	ME	16	0.339	1.028 (0.973, 1.085)	13.147	0.515			
	WM	16	0.055	1.044 (0.999, 1.090)					
	SM	16	0.372	1.060 (0.936, 1.201)					
	W‐Mode	16	0.064	1.043 (1.001, 1.086)					
Carbohydrate									
Galactonate	IVW	7	0.022	1.209 (1.028, 1.423)	9.704	0.138	0.042	0.315	Increase
	ME	7	0.900	0.973 (0.642, 1.472)	7.771	0.169			
	WM	7	0.007	1.275 (1.067, 1.524)					
	SM	7	0.080	1.299 (1.018, 1.659)					
	W‐Mode	7	0.063	1.286 (1.035, 1.597)					
Cofactors and vitamins									
Gulonate	IVW	9	0.029	1.158 (1.015, 1.320)	4.222	0.837	−0.033	0.135	Increase
	ME	9	0.053	1.591 (1.076, 2.353)	1.361	0.987			
	WM	9	0.050	1.187 (1.000, 1.408)					
	SM	9	0.224	1.209 (0.911, 1.604)					
	W‐Mode	9	0.206	1.199 (0.926, 1.552)					
Ascorbic acid 2‐sulfate	IVW	11	0.018	0.875 (0.784, 0.977)	5.167	0.880	0.000	1.000	Decrease
	ME	11	0.524	0.875 (0.591, 1.297)	5.167	0.819			
	WM	11	0.031	0.852 (0.737, 0.985)					
	SM	11	0.169	0.840 (0.666, 1.058)					
	W‐Mode	11	0.104	0.823 (0.664, 1.019)					
Bilirubin (E, E)	IVW	10	0.042	1.048 (1.002, 1.096)	2.004	0.991	0.006	0.498	Increase
	ME	10	0.374	1.031 (0.968, 1.098)	1.501	0.993			
	WM	10	0.111	1.040 (0.991, 1.092)					
	SM	10	0.469	1.057 (0.916, 1.220)					
	W‐Mode	10	0.146	1.039 (0.991, 1.090)					
Lipid									
Docosahexaenoate	IVW	8	0.028	1.168 (1.017, 1.341)	2.435	0.932	−0.008	0.772	Increase
	ME	8	0.434	1.269 (0.727, 2.217)	2.343	0.886			
	WM	8	0.247	1.112 (0.929, 1.331)					
	SM	8	0.476	1.109 (0.847, 1.453)					
	W‐Mode	8	0.488	1.104 (0.847, 1.440)					
5‐Hydroxyhexanoate	IVW	9	0.005	1.192 (1.055, 1.347)	2.201	0.974	0.006	0.779	Increase
	ME	9	0.444	1.141 (0.830, 1.569)	2.116	0.953			
	WM	9	0.027	1.182 (1.019, 1.371)					
	SM	9	0.123	1.218 (0.973, 1.523)					
	W‐Mode	9	0.105	1.229 (0.985, 1.532)					
Docosatrienoate (22:3n3)	IVW	11	0.037	0.889 (0.797, 0.993)	17.638	0.061	−0.027	0.110	Decrease
	ME	11	0.604	1.062 (0.852, 1.324)	13.080	0.159			
	WM	11	0.442	0.956 (0.853, 1.072)					
	SM	11	0.720	0.950 (0.724, 1.247)					
	W‐Mode	11	0.610	0.968 (0.859, 1.091)					
Hyocholate	IVW	3	0.017	1.250 (1.041, 1.502)	2.449	0.294	0.001	0.985	Increase
	ME	3	0.575	1.243 (0.723, 2.138)	2.447	0.118			
	WM	3	0.025	1.303 (1.035, 1.642)					
	SM	3	0.150	1.371 (1.045, 1.798)					
	W‐Mode	3	0.169	1.366 (1.023, 1.825)					
1‐Oleoyl‐GPI (18:1)	IVW	13	0.027	1.118 (1.013, 1.234)	8.020	0.784	0.006	0.788	Increase
	ME	13	0.684	1.071 (0.777, 1.477)	7.944	0.718			
	WM	13	0.020	1.171 (1.025, 1.336)					
	SM	13	0.189	1.161 (0.941, 1.433)					
	W‐Mode	13	0.188	1.161 (0.941, 1.432)					
Chiro‐inositol	IVW	6	0.012	1.161 (1.034, 1.304)	3.943	0.558	−0.026	0.527	Increase
	ME	6	0.292	1.402 (0.812, 2.419)	3.464	0.483			
	WM	6	0.039	1.164 (1.008, 1.343)					
	SM	6	0.236	1.172 (0.930, 1.477)					
	W‐Mode	6	0.194	1.170 (0.953, 1.437)					
5Alpha‐androstan‐3alpha,17beta‐diol disulfate	IVW	16	0.008	1.123 (1.031, 1.223)	17.182	0.308	−0.017	0.199	Increase
	ME	16	0.040	1.296 (1.035, 1.623)	15.208	0.364			
	WM	16	0.067	1.109 (0.993, 1.239)					
	SM	16	0.308	1.105 (0.918, 1.331)					
	W‐Mode	16	0.228	1.105 (0.946, 1.292)					
4‐Hydroxy‐2‐oxoglutaric acid	IVW	12	0.010	0.871 (0.784, 0.967)	7.479	0.759	0.005	0.738	Decrease
	ME	12	0.051	0.872 (0.761, 1.000)	7.361	0.691			
	WM	12	0.183	0.838 (0.657, 1.068)					
	SM	12	0.194	0.847 (0.669, 1.072)					
	W‐Mode	12	0.187	0.855 (0.686, 1.064)					
Glycohyocholate	IVW	7	0.040	1.138 (1.006, 1.286)	2.464	0.872	0.012	0.522	Increase
	ME	7	0.872	1.028 (0.750, 1.407)	1.990	0.851			
	WM	7	0.143	1.122 (0.962, 1.310)					
	SM	7	0.602	1.065 (0.850, 1.335)					
	W‐Mode	7	0.453	1.093 (0.879, 1.360)					
1‐(1‐Enyl‐palmitoyl)‐2‐oleoyl‐GPE (p‐16:0/18:1)	IVW	13	0.033	1.114 (1.009, 1.232)	6.383	0.896	−0.007	0.698	Increase
	ME	13	0.245	1.166 (0.912, 1.491)	6.224	0.858			
	WM	13	0.203	1.089 (0.955, 1.242)					
	SM	13	0.644	1.054 (0.847, 1.312)					
	W‐Mode	13	0.561	1.062 (0.872, 1.293)					
Glycosyl‐*N*‐tricosanoyl‐sphingadienine (d18:2/23:0)	IVW	8	0.001	0.821 (0.729, 0.924)	6.792	0.451	0.008	0.711	Decrease
	ME	8	0.240	0.759 (0.501, 1.149)	6.624	0.357			
	WM	8	0.004	0.792 (0.675, 0.930)					
	SM	8	0.087	0.754 (0.571, 0.996)					
	W‐Mode	8	0.095	0.759 (0.574, 1.004)					
Dodecenedioate (C12:1‐DC)	IVW	12	0.003	1.146 (1.046, 1.255)	8.592	0.660	0.008	0.634	Increase
	ME	12	0.543	1.082 (0.846, 1.384)	8.351	0.595			
	WM	12	0.093	1.110 (0.983, 1.255)					
	SM	12	0.109	1.217 (0.976, 1.517)					
	W‐Mode	12	0.146	1.106 (0.975, 1.254)					
11Beta‐hydroxyetiocholanolone glucuronide	IVW	10	0.007	1.159 (1.041, 1.291)	7.949	0.539	−0.013	0.433	Increase
	ME	10	0.094	1.276 (0.992, 1.641)	7.269	0.508			
	WM	10	0.022	1.184 (1.025, 1.368)					
	SM	10	0.179	1.183 (0.944, 1.483)					
	W‐Mode	10	0.135	1.195 (0.966, 1.479)					
4‐Methylhexanoylglutamine	IVW	3	0.008	0.793 (0.667, 0.942)	1.114	0.573	0.016	0.720	Decrease
	ME	3	0.392	0.714 (0.448, 1.139)	0.892	0.345			
	WM	3	0.073	0.826 (0.670, 1.018)					
	SM	3	0.305	0.837 (0.648, 1.080)					
	W‐Mode	3	0.241	0.833 (0.670, 1.035)					
Tetrahydrocortisol glucuronide	IVW	16	0.038	1.106 (1.006, 1.216)	15.533	0.414	−0.008	0.460	Increase
	ME	16	0.293	1.072 (0.941, 1.221)	14.917	0.384			
	WM	16	0.126	1.187 (0.965, 1.460)					
	SM	16	0.892	1.016 (0.814, 1.267)					
	W‐Mode	16	0.662	1.043 (0.868, 1.253)					
Glycerol	IVW	5	0.008	1.317 (1.076, 1.612)	1.027	0.906	−0.005	0.955	Increase
	ME	5	0.716	1.383 (0.282, 6.791)	1.023	0.796			
	WM	5	0.100	1.246 (0.958, 1.621)					
	SM	5	0.291	1.222 (0.885, 1.688)					
	W‐Mode	5	0.325	1.219 (0.862, 1.723)					
Ceramide (d18:1/16:0)	IVW	13	0.005	0.859 (0.773, 0.954)	9.155	0.690	0.021	0.161	Decrease
	ME	13	0.026	0.723 (0.564, 0.926)	6.900	0.807			
	WM	13	0.013	0.837 (0.728, 0.962)					
	SM	13	0.143	0.821 (0.642, 1.051)					
	W‐Mode	13	0.131	0.821 (0.647, 1.042)					
Glycocholate	IVW	17	0.041	0.910 (0.831, 0.996)	7.555	0.961	−0.010	0.479	Decrease
	ME	17	0.911	0.986 (0.779, 1.249)	7.029	0.957			
	WM	17	0.052	0.884 (0.781, 1.001)					
	SM	17	0.266	0.876 (0.699, 1.097)					
	W‐Mode	17	0.221	0.876 (0.714, 1.074)					
Nucleotide									
Inosine 5′‐monophosphate	IVW	10	0.041	0.910 (0.831, 0.996)	6.449	0.694	0.003	0.877	Decrease
	ME	10	0.386	0.893 (0.702, 1.137)	6.423	0.600			
	WM	10	0.158	0.914 (0.807, 1.035)					
	SM	10	0.116	0.827 (0.667, 1.024)					
	W‐Mode	10	0.113	0.829 (0.673, 1.022)					
Cytidine	IVW	12	0.020	0.880 (0.790, 0.980)	10.673	0.471	0.024	0.137	Decrease
	ME	12	0.029	0.750 (0.600, 0.936)	8.052	0.624			
	WM	12	0.302	0.923 (0.792, 1.075)					
	SM	12	0.218	0.852 (0.671, 1.083)					
	W‐Mode	12	0.375	0.916 (0.761, 1.103)					
Partially characterized molecules									
Bilirubin degradation product, C17H20N2O5 (2)	IVW	10	0.041	1.070 (1.003, 1.141)	4.501	0.875	0.014	0.308	Increase
	ME	10	0.792	1.016 (0.907, 1.137)	3.315	0.913			
	WM	10	0.091	1.062 (0.990, 1.140)					
	SM	10	0.315	1.096 (0.926, 1.296)					
	W‐Mode	10	0.148	1.061 (0.986, 1.141)					
Peptide									
Gamma‐glutamyl‐2‐aminobutyrate	IVW	11	0.027	0.884 (0.792, 0.986)	6.500	0.772	−0.005	0.803	Decrease
	ME	11	0.651	0.922 (0.656, 1.296)	6.433	0.696			
	WM	11	0.025	0.849 (0.736, 0.980)					
	SM	11	0.125	0.836 (0.677, 1.031)					
	W‐Mode	11	0.154	0.836 (0.666, 1.050)					
Phenylacetylglutamate	IVW	9	0.040	1.107 (1.005, 1.220)	4.720	0.787	−0.018	0.491	Increase
	ME	9	0.232	1.242 (0.897, 1.719)	4.192	0.757			
	WM	9	0.049	1.138 (1.000, 1.294)					
	SM	9	0.159	1.173 (0.959, 1.434)					
	W‐Mode	9	0.131	1.151 (0.977, 1.356)					
4‐Hydroxyphenylacetylglutamine	IVW	12	0.026	0.867 (0.765, 0.983)	15.895	0.145	−0.041	0.148	Decrease
	ME	12	0.332	1.327 (0.770, 2.287)	12.755	0.238			
	WM	12	0.360	0.932 (0.803, 1.083)					
	SM	12	0.859	1.025 (0.786, 1.336)					
	W‐Mode	12	0.973	0.996 (0.794, 1.250)					
3‐Hydroxyphenylacetoylglutamine	IVW	2	0.029	0.779 (0.623, 0.974)	0.380	0.537			Decrease
Xenobiotics									
Methyl vanillate sulfate	IVW	8	0.028	0.880 (0.785, 0.987)	9.074	0.247	0.019	0.627	Decrease
	ME	8	0.303	0.798 (0.539, 1.182)	8.694	0.192			
	WM	8	0.111	0.892 (0.774, 1.027)					
	SM	8	0.565	0.929 (0.731, 1.181)					
	W‐Mode	8	0.631	0.942 (0.747, 1.189)					
5‐Hydroxymethyl‐2‐furoylcarnitine	IVW	8	0.015	1.136 (1.025, 1.260)	5.350	0.617	0.051	0.368	Increase
	ME	8	0.514	0.722 (0.288, 1.810)	4.403	0.622			
	WM	8	0.013	1.188 (1.037, 1.361)					
	SM	8	0.128	1.203 (0.975, 1.483)					
	W‐Mode	8	0.124	1.201 (0.978, 1.474)					
Amino acid/amino acid									
Spermidine to ornithine ratio	IVW	5	0.018	1.196 (1.031, 1.388)	1.946	0.746	−0.004	0.873	Increase
	ME	5	0.296	1.226 (0.893, 1.684)	1.916	0.590			
	WM	5	0.042	1.213 (1.007, 1.462)					
	SM	5	0.173	1.240 (0.961, 1.599)					
	W‐Mode	5	0.163	1.226 (0.970, 1.548)					
Glutamate to cysteine ratio	IVW	12	0.018	1.138 (1.023, 1.266)	8.242	0.691	0.003	0.904	Increase
	ME	12	0.544	1.115 (0.794, 1.566)	8.227	0.607			
	WM	12	0.237	1.089 (0.945, 1.255)					
	SM	12	0.586	1.076 (0.834, 1.388)					
	W‐Mode	12	0.586	1.076 (0.834, 1.388)					
Cysteine to alanine ratio	IVW	13	0.005	1.164 (1.048, 1.295)	10.058	0.611	−0.009	0.615	Increase
	ME	13	0.139	1.242 (0.952, 1.621)	9.790	0.549			
	WM	13	0.212	1.096 (0.949, 1.265)					
	SM	13	0.954	1.008 (0.773, 1.315)					
	W‐Mode	13	0.941	1.010 (0.777, 1.313)					
Proline to glutamate ratio	IVW	9	0.035	0.883 (0.786, 0.991)	7.492	0.485	−0.011	0.609	Decrease
	ME	9	0.669	0.942 (0.723, 1.227)	7.197	0.409			
	WM	9	0.447	0.941 (0.804, 1.101)					
	SM	9	0.775	0.963 (0.748, 1.238)					
	W‐Mode	9	0.856	0.981 (0.806, 1.195)					
Energy/carbohydrate									
Phosphate to *N*‐acetylneuraminate	IVW	15	0.049	1.107 (1.001, 1.224)	6.961	0.936	0.005	0.670	Increase
	ME	15	0.625	1.059 (0.847, 1.324)	6.771	0.914			
	WM	15	0.209	1.087 (0.955, 1.237)					
	SM	15	0.709	1.047 (0.828, 1.323)					
	W‐Mode	15	0.636	1.059 (0.840, 1.334)					
Lipid/cofactors and vitamins									
Cholate to bilirubin (Z, Z) ratio	IVW	10	0.048	0.917 (0.841, 0.999)	5.112	0.824	−0.007	0.607	Decrease
	ME	10	0.679	0.960 (0.795, 1.159)	4.826	0.776			
	WM	10	0.079	0.908 (0.816, 1.011)					
	SM	10	0.338	0.910 (0.759, 1.092)					
	W‐Mode	10	0.149	0.910 (0.810, 1.023)					
Lipid/lipid									
Glycolithocholate to glycolithocholate sulfate ratio	IVW	14	0.008	0.902 (0.835, 0.974)	8.371	0.819	−0.015	0.497	Decrease
	ME	14	0.829	0.974 (0.775, 1.226)	7.879	0.794			
	WM	14	0.067	0.909 (0.820, 1.007)					
	SM	14	0.302	0.915 (0.778, 1.076)					
	W‐Mode	14	0.238	0.922 (0.810, 1.049)					
Nucleotide/amino acid									
ADP to tyrosine ratio	IVW	8	0.042	1.106 (1.003, 1.218)	4.420	0.730	0.019	0.344	Increase
	ME	8	0.899	0.984 (0.771, 1.255)	3.366	0.762			
	WM	8	0.148	1.099 (0.967, 1.248)					
	SM	8	0.254	1.132 (0.931, 1.376)					
	W‐Mode	8	0.267	1.120 (0.931, 1.347)					
AMP to methionine ratio	IVW	6	0.006	0.807 (0.693, 0.940)	3.216	0.667	−0.031	0.209	Decrease
	ME	6	0.702	1.094 (0.714, 1.677)	0.982	0.912			
	WM	6	0.031	0.806 (0.662, 0.981)					
	SM	6	0.204	0.809 (0.609, 1.075)					
	W‐Mode	6	0.207	0.792 (0.578, 1.085)					
AMP to isoleucine ratio	IVW	6	0.002	0.777 (0.664, 0.909)	4.015	0.547	−0.027	0.341	Decrease
	ME	6	0.984	1.005 (0.614, 1.647)	2.852	0.583			
	WM	6	0.056	0.818 (0.666, 1.005)					
	SM	6	0.243	0.814 (0.600, 1.104)					
	W‐Mode	6	0.214	0.820 (0.623, 1.078)					
Nucleotide/carbohydrate									
ADP to mannitol to sorbitol ratio	IVW	7	0.034	1.164 (1.012, 1.340)	9.494	0.148	0.035	0.197	Increase
	ME	7	0.693	0.934 (0.681, 1.283)	6.585	0.253			
	WM	7	0.067	1.163 (0.990, 1.366)					
	SM	7	0.178	1.245 (0.939, 1.651)					
	W‐Mode	7	0.173	1.251 (0.942, 1.662)					
Nucleotide/cofactors and vitamins									
AMP to flavin adenine dinucleotide (FAD) ratio	IVW	6	0.044	0.895 (0.804, 0.997)	5.449	0.364	0.020	0.525	Decrease
	ME	6	0.238	0.815 (0.610, 1.088)	4.861	0.302			
	WM	6	0.113	0.898 (0.785, 1.026)					
	SM	6	0.138	0.827 (0.670, 1.021)					
	W‐Mode	6	0.238	0.894 (0.759, 1.053)					
Nucleotide/nucleotide									
ADP to AMP ratio	IVW	12	0.003	1.134 (1.045, 1.231)	6.809	0.814	−0.016	0.368	Increase
	ME	12	0.082	1.261 (0.996, 1.596)	5.920	0.822			
	WM	12	0.026	1.130 (1.015, 1.259)					
	SM	12	0.155	1.146 (0.962, 1.366)					
	W‐Mode	12	0.150	1.141 (0.965, 1.348)					
Unknown									
X‐12839	IVW	12	0.002	0.881 (0.814, 0.955)	8.813	0.639	0.011	0.450	Decrease
	ME	12	0.089	0.814 (0.657, 1.009)	8.195	0.610			
	WM	12	0.004	0.852 (0.765, 0.950)					
	SM	12	0.071	0.839 (0.706, 0.996)					
	W‐Mode	12	0.074	0.844 (0.713, 0.999)					
X‐13695	IVW	6	0.014	0.836 (0.725, 0.965)	4.757	0.446	0.022	0.378	Decrease
	ME	6	0.123	0.719 (0.516, 1.002)	3.777	0.437			
	WM	6	0.042	0.826 (0.687, 0.993)					
	SM	6	0.132	0.761 (0.565, 1.025)					
	W‐Mode	6	0.148	0.763 (0.559, 1.040)					
X‐18886	IVW	7	0.017	0.848 (0.742, 0.971)	7.079	0.314	−0.030	0.152	Decrease
	ME	7	0.529	1.130 (0.792, 1.613)	4.232	0.517			
	WM	7	0.023	0.815 (0.684, 0.972)					
	SM	7	0.087	0.739 (0.553, 0.988)					
	W‐Mode	7	0.128	0.773 (0.581, 1.029)					
X‐18887	IVW	5	0.038	0.851 (0.731, 0.991)	3.117	0.538	0.019	0.494	Decrease
	ME	5	0.260	0.709 (0.436, 1.153)	2.514	0.473			
	WM	5	0.116	0.850 (0.694, 1.041)					
	SM	5	0.298	0.849 (0.650, 1.110)					
	W‐Mode	5	0.299	0.849 (0.649, 1.111)					
X‐18888	IVW	7	0.009	1.182 (1.043, 1.340)	6.642	0.355	0.025	0.319	Increase
	ME	7	0.835	0.957 (0.645, 1.420)	5.337	0.376			
	WM	7	0.073	1.161 (0.986, 1.366)					
	SM	7	0.309	1.134 (0.909, 1.415)					
	W‐Mode	7	0.246	1.150 (0.930, 1.422)					
X‐21353	IVW	11	0.016	1.117 (1.021, 1.223)	7.741	0.654	0.008	0.579	Increase
	ME	11	0.584	1.060 (0.867, 1.296)	7.410	0.595			
	WM	11	0.139	1.093 (0.972, 1.230)					
	SM	11	0.370	1.102 (0.900, 1.348)					
	W‐Mode	11	0.163	1.095 (0.973, 1.232)					
X‐21821	IVW	4	0.038	0.827 (0.692, 0.989)	1.163	0.762	−0.020	0.625	Decrease
	ME	4	0.993	1.003 (0.505, 1.991)	0.837	0.658			
	WM	4	0.147	0.859 (0.700, 1.055)					
	SM	4	0.376	0.864 (0.654, 1.140)					
	W‐Mode	4	0.365	0.862 (0.656, 1.133)					
X‐21834	IVW	9	0.041	1.133 (1.005, 1.277)	1.421	0.994	0.007	0.730	Increase
	ME	9	0.709	1.069 (0.762, 1.500)	1.292	0.989			
	WM	9	0.098	1.137 (0.977, 1.323)					
	SM	9	0.257	1.152 (0.918, 1.445)					
	W‐Mode	9	0.279	1.146 (0.910, 1.443)					
X‐25217	IVW	10	0.038	0.916 (0.843, 0.995)	9.261	0.414	0.000	0.999	Decrease
	ME	10	0.430	0.916 (0.744, 1.127)	9.261	0.321			
	WM	10	0.039	0.887 (0.791, 0.994)					
	SM	10	0.199	0.870 (0.714, 1.060)					
	W‐Mode	10	0.192	0.868 (0.714, 1.057)					
X‐25810	IVW	16	0.034	0.905 (0.825, 0.993)	14.336	0.500	−0.005	0.701	Decrease
	ME	16	0.560	0.939 (0.764, 1.154)	14.180	0.436			
	WM	16	0.117	0.902 (0.793, 1.026)					
	SM	16	0.223	0.884 (0.731, 1.069)					
	W‐Mode	16	0.338	0.906 (0.745, 1.102)					

Abbreviations: ADP, adenosine 5′‐diphosphate; AMP, adenosine 5′‐monophosphate; CI, confidence interval; DMTPA, 2,3‐Dihydroxy‐5‐methylthio‐4‐pentenoate; FAD, Flavin adenine dinucleotide; IVW, inverse variance weighted; ME, MR‐Egger; OR, odds ratio; SM, simple mode; SNPs, single‐nucleotide polymorphisms; W‐mode, weighted mode; WM, weighted median.

### 3.2 Sensitivity Analysis

**Figure 4:**
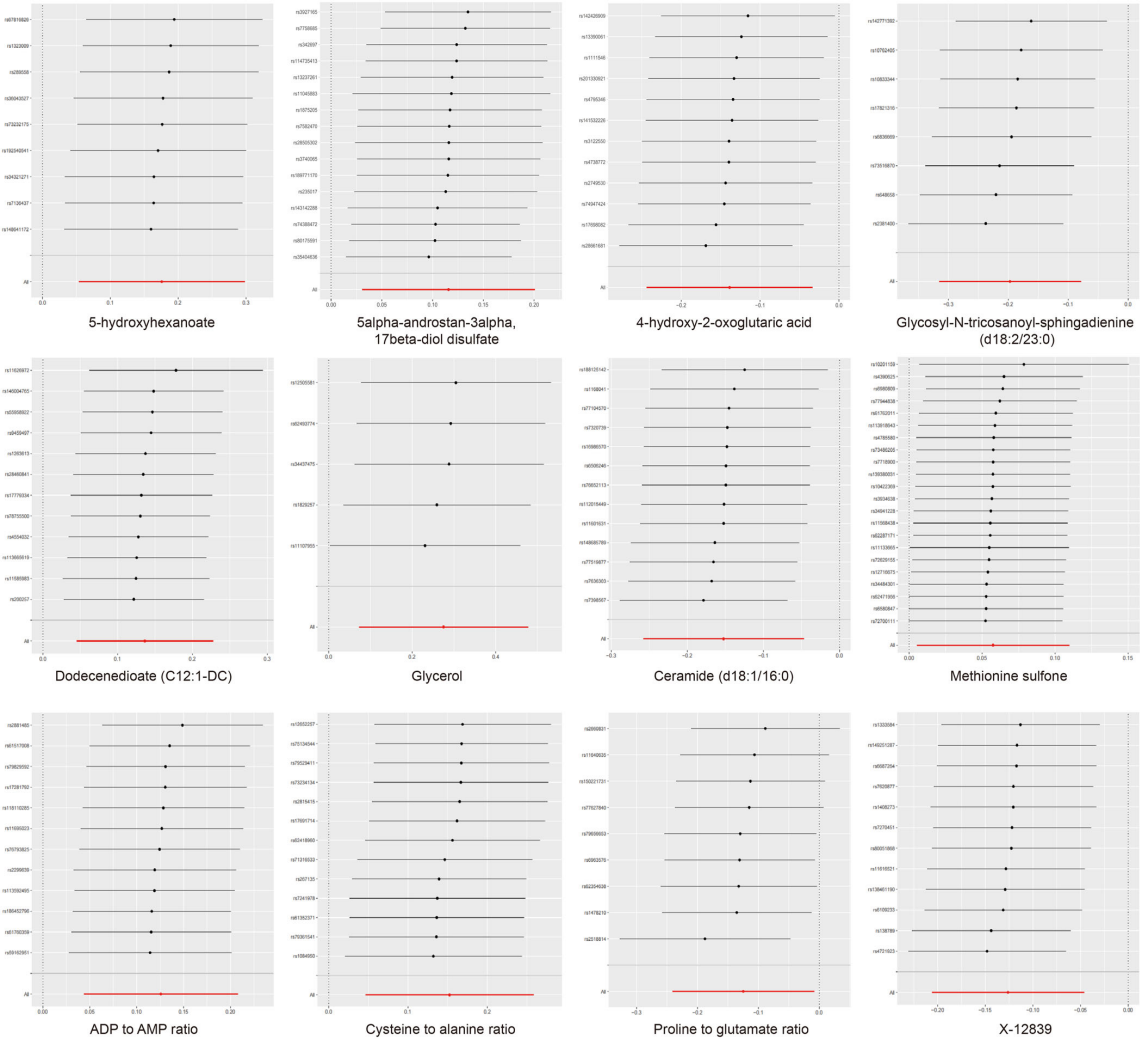
Leave‐one‐out plots illustrating the causal associations between blood metabolites and ASD. Abbreviations: ADP, adenosine 5′‐diphosphate; AMP, adenosine 5′‐monophosphate; ASD, autism spectrum disorder.

**Figure 5:**
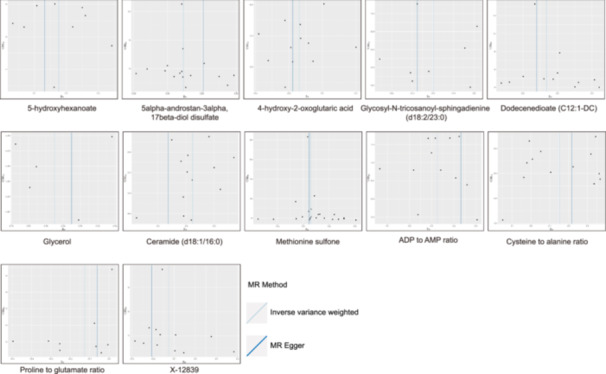
Funnel plots to visualize the overall heterogeneity of MR estimates for causal associations between blood metabolites and ASD. Abbreviations: ADP, adenosine 5′‐diphosphate; AMP, adenosine 5′‐monophosphate; ASD, autism spectrum disorder.

To assess the rigidity of the above results, a series of sensitivity analyses were conducted, including Cochran's Q test, the MR‐Egger intercept test, LOO analysis, and funnel plot [46]. As a result, 9 metabolites and 3 ratio that satisfied the stringent screening criteria were identified as potential candidates (Table 1), including 5‐hydroxyhexanoate (OR = 1.192, 95% CI: 1.055–1.347, *p* = 0.005), 5alpha‐androstan‐3alpha,17beta‐diol disulfate (OR = 1.123, 95% CI: 1.031–1.223, *p* = 0.008), 4‐hydroxy‐2‐oxoglutaric acid (OR = 0.871, 95% CI: 0.784–0.967, *p* = 0.010), Glycosyl‐*N*‐tricosanoyl‐sphingadienine (d18:2/23:0) (OR = 0.821, 95% CI: 0.729–0.924, *p* = 0.001), C12:1‐DC (OR = 1.146, 95% CI: 1.046–1.255, *p* = 0.003), Glycerol (OR = 1.317, 95% CI: 1.076–1.612, *p* = 0.008), Ceramide (d18:1/16:0) (OR = 0.859, 95% CI: 0.773–0.954, *p* = 0.005), Methionine sulfone (OR = 1.059, 95% CI: 1.005–1.116, *p* = 0.031), ADP to AMP ratio (OR = 1.134, 95% CI: 1.045–1.231, *p* = 0.003), Cysteine to alanine ratio (OR = 1.164, 95% CI: 1.048–1.295, *p* = 0.005), Proline to glutamate ratio (OR = 0.883, 95% CI: 0.786–0.991, *p* = 0.035), X‐12839 (OR = 0.881, 95% CI: 0.814–0.955, *p* = 0.002). As shown in Table 1, these potential candidates showed no heterogeneity or pleiotropy (*p* > 0.05). For these metabolites, LOO analyses revealed that the estimates by a single SNP in Figure 4 and Figure S5 and the funnel plot were symmetrical (Figure 5 and Figure S6). These findings provide crucial insights into metabolic alterations in ASD and may contribute to the development of future diagnostic and therapeutic strategies.

### 3.3 Replication Analysis

**Figure 6:**
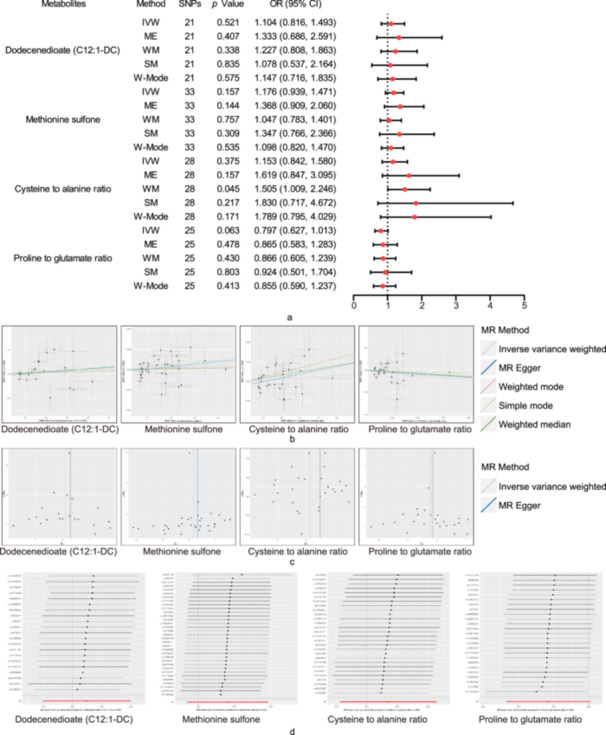
Replication analysis of the causal relationship between blood and ASD. (a) Forest plot (b) Scatter plots (c) Funnel plots (d) Plots of LOO. Abbreviations: ASD, autism spectrum disorder; CI, confidence interval; IVW, inverse variance weighted; LOO, leave‐one‐out; ME, MR‐Egger; OR, odds ratio; SM, simple mode; SNPs, single‐nucleotide polymorphisms; WM, weighted median; W‐Mode, weighted mode.

To further confirm our results, replication analysis was performed using ASD data from the FinnGen consortium. Similar trends were also observed for C12:1‐DC, methionine sulfone, cysteine to alanine ratio, and proline to glutamate ratio in FinnGen for ASD, although the findings were not statistically significant owing to a considerable disparity in sample sizes [46] (Tables 1 and 2, Figures 2 and 6, Figure S7).

**Table 2:** Replication analysis of the causal relationship between blood and ASD.

Metabolites	Method	SNPs	OR		Heterogeneity		Pleiotropy	
			*p* Value	OR (95% CI)	*Q*	*p* Value	Egger intercept	*p* Value
5‐Hydroxyhexanoate	IVW	19	0.587	0.922 (0.689, 1.234)	12.384	0.827	0.011	0.785
	ME	19	0.611	0.862 (0.492, 1.511)	12.308	0.781		
	WM	19	0.598	0.887 (0.568, 1.386)				
	SM	19	0.620	0.842 (0.431, 1.645)				
	W‐Mode	19	0.613	0.868 (0.507, 1.487)				
5Alpha‐androstan‐3alpha,17beta‐diol disulfate	IVW	28	0.686	1.054 (0.818, 1.357)	32.006	0.232	−0.002	0.946
	ME	28	0.813	1.073 (0.604, 1.905)	32.000	0.193		
	WM	28	0.318	0.832 (0.580, 1.194)				
	SM	28	0.333	0.666 (0.297, 1.494)				
	W‐Mode	28	0.401	0.699 (0.307, 1.592)				
4‐Hydroxy‐2‐oxoglutaric acid	IVW	28	0.173	1.226 (0.914, 1.643)	28.148	0.403	−0.052	0.155
	ME	28	0.061	1.789 (1.000, 3.202)	26.003	0.463		
	WM	28	0.132	1.390 (0.906, 2.134)				
	SM	28	0.193	1.825 (0.755, 4.410)				
	W‐Mode	28	0.155	1.754 (0.826, 3.725)				
Glycosyl‐*N*‐tricosanoyl‐sphingadienine (d18:2/23:0)	IVW	25	0.187	1.187 (0.920, 1.530)	25.365	0.386	−0.030	0.436
	ME	25	0.209	1.461 (0.823, 2.594)	24.690	0.366		
	WM	25	0.472	1.162 (0.771, 1.752)				
	SM	25	0.588	0.826 (0.418, 1.634)				
	W‐Mode	25	0.737	0.892 (0.462, 1.723)				
C12:1‐DC	IVW	21	0.521	1.104 (0.816, 1.493)	22.196	0.330	−0.030	0.538
	ME	21	0.407	1.333 (0.686, 2.591)	21.746	0.297		
	WM	21	0.338	1.227 (0.808, 1.863)				
	SM	21	0.835	1.078 (0.537, 2.164)				
	W‐Mode	21	0.575	1.147 (0.716, 1.835)				
Glycerol	IVW	23	0.592	0.907 (0.635, 1.296)	37.583	0.020	−0.058	0.112
	ME	23	0.370	1.284 (0.752, 2.192)	33.221	0.044		
	WM	23	0.463	1.178 (0.761, 1.821)				
	SM	23	0.431	1.494 (0.560, 3.983)				
	W‐Mode	23	0.312	1.224 (0.835, 1.796)				
Ceramide (d18:1/16:0)	IVW	26	0.779	0.959 (0.715, 1.286)	25.422	0.439	0.036	0.384
	ME	26	0.366	0.719 (0.357, 1.449)	24.616	0.427		
	WM	26	0.949	1.014 (0.666, 1.543)				
	SM	26	0.969	1.018 (0.418, 2.479)				
	W‐Mode	26	0.698	1.165 (0.543, 2.499)				
Methionine sulfone	IVW	33	0.157	1.176 (0.939, 1.471)	43.115	0.091	−0.026	0.391
	ME	33	0.144	1.368 (0.909, 2.060)	42.090	0.088		
	WM	33	0.757	1.047 (0.783, 1.401)				
	SM	33	0.309	1.347 (0.766, 2.366)				
	W‐Mode	33	0.535	1.098 (0.820, 1.470)				
ADP to AMP ratio	IVW	25	0.802	0.972 (0.779, 1.213)	21.090	0.633	−0.009	0.818
	ME	25	0.925	1.024 (0.627, 1.672)	21.036	0.579		
	WM	25	0.704	1.064 (0.773, 1.465)				
	SM	25	0.563	1.191 (0.663, 2.140)				
	W‐Mode	25	0.735	1.093 (0.657, 1.819)				
Cysteine to alanine ratio	IVW	28	0.375	1.153 (0.842, 1.580)	36.048	0.114	−0.051	0.252
	ME	28	0.157	1.619 (0.847, 3.095)	34.239	0.129		
	WM	28	0.045	1.505 (1.009, 2.246)				
	SM	28	0.217	1.830 (0.717, 4.672)				
	W‐Mode	28	0.171	1.789 (0.795, 4.029)				
Proline to glutamate ratio	IVW	25	0.063	0.797 (0.627, 1.013)	25.551	0.376	−0.019	0.611
	ME	25	0.478	0.865 (0.583, 1.283)	25.260	0.337		
	WM	25	0.43	0.866 (0.605, 1.239)				
	SM	25	0.803	0.924 (0.501, 1.704)				
	W‐Mode	25	0.413	0.855 (0.590, 1.237)				
X‐12839	IVW	28	0.718	0.956 (0.750, 1.219)	35.486	0.127	−0.025	0.528
	ME	28	0.693	1.112 (0.659, 1.878)	34.935	0.113		
	WM	28	0.986	1.003 (0.739, 1.361)				
	SM	28	0.836	1.060 (0.612, 1.836)				
	W‐Mode	28	0.945	1.018 (0.622, 1.665)				

Abbreviations: ADP, adenosine 5′‐diphosphate; AMP, adenosine 5′‐monophosphate; C12:1‐DC, dodecenedioate.

### 3.4 Confounding Analysis

Confounding analysis revealed that SNPs associated with potential candidates were independent of any confounding factors (Table S1). However, confounding factors included galactonate, argininate, DMTPA, X‐12839, X‐25810, *N*‐acetyl‐l‐glutamine, N6‐methyllysine, and AMP to FAD ratio (Table S2).

### 3.5 Reserve Analysis

**Figure 7:**
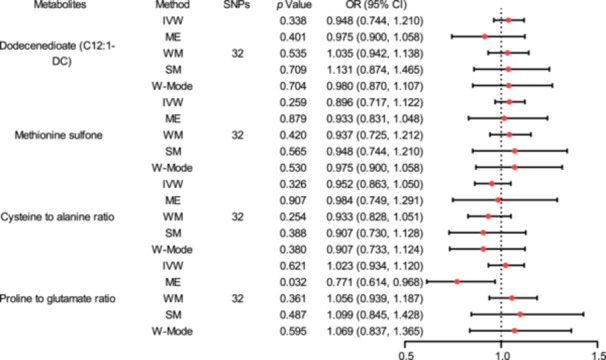
Reserve analysis of the final identified blood metabolites. Abbreviations: CI, confidence interval; IVW, inverse variance weighted; ME, MR‐Egger; OR, odds ratio; SM, simple mode; SNPs, single‐nucleotide polymorphisms; WM, weighted median; W‐Mode, weighted mode.

Reserve TSMR analysis was used to investigate the effect of ASD on blood metabolites. The data presented in Table 3, Figure 7, and Figure S8 highlight the influence of ASD on blood metabolites, as revealed through MR analysis using ASD data sourced from Jakob Grove et al. [16], along with results from sensitivity assessments. Across all employed reverse analysis methodologies, there was no substantial evidence suggesting a causal relationship between blood metabolites and the risk of ASD among the 12 positively identified metabolites in the preliminary screening (*p* > 0.05). The ORs observed were exceedingly close to 1, underscoring the minimal effect of ASD on blood metabolite levels.

**Table 3:** MR estimates and sensitivity analyses for the effect of ASD on blood metabolites.

Exposure	Metabolites	Method	SNPs	OR		Heterogeneity		Pleiotropy	
				*p* Value	OR (95% CI)	*Q*	*p* Value	Egger intercept	*p* Value
ASD	C12:1‐DC	IVW	32	0.338	0.948 (0.744, 1.210)	19.634	0.943	0.012	0.206
		ME	32	0.401	0.975 (0.900, 1.058)	17.960	0.959		
		WM	32	0.535	1.035 (0.942, 1.138)				
		SM	32	0.709	1.131 (0.874, 1.465)				
		W‐Mode	32	0.704	0.980 (0.870, 1.107)				
	Methionine sulfone	IVW	32	0.259	0.896 (0.717, 1.122)	27.878	0.627	0.003	0.781
		ME	32	0.879	0.933 (0.831, 1.048)	27.799	0.581		
		WM	32	0.420	0.937 (0.725, 1.212)				
		SM	32	0.565	0.948 (0.744, 1.210)				
		W‐Mode	32	0.530	0.975 (0.900, 1.058)				
	Cysteine to alanine ratio	IVW	32	0.326	0.952 (0.863, 1.050)	48.678	0.023	−0.003	0.801
		ME	32	0.907	0.984 (0.749, 1.291)	48.573	0.017		
		WM	32	0.254	0.933 (0.828, 1.051)				
		SM	32	0.388	0.907 (0.730, 1.128)				
		W‐Mode	32	0.380	0.907 (0.733, 1.124)				
	Proline to glutamate ratio	IVW	32	0.621	1.023 (0.934, 1.120)	43.898	0.062	0.028	0.014
		ME	32	0.032	0.771 (0.614, 0.968)	35.702	0.218		
		WM	32	0.361	1.056 (0.939, 1.187)				
		SM	32	0.487	1.099 (0.845, 1.428)				
		W‐Mode	32	0.595	1.069 (0.837, 1.365)				

Abbreviations: ASD, autism spectrum disorder; CI, confidence interval; C12:1‐DC, dodecenedioate; IVW, inverse variance weighted; ME, MR‐Egger; SM, simple mode; SNPs, single‐nucleotide polymorphisms; WM, weighted median; W‐Mode, weighted mode.

### 3.6 Metabolic Pathways and Network Pharmacology Analysis

**Figure 8:**
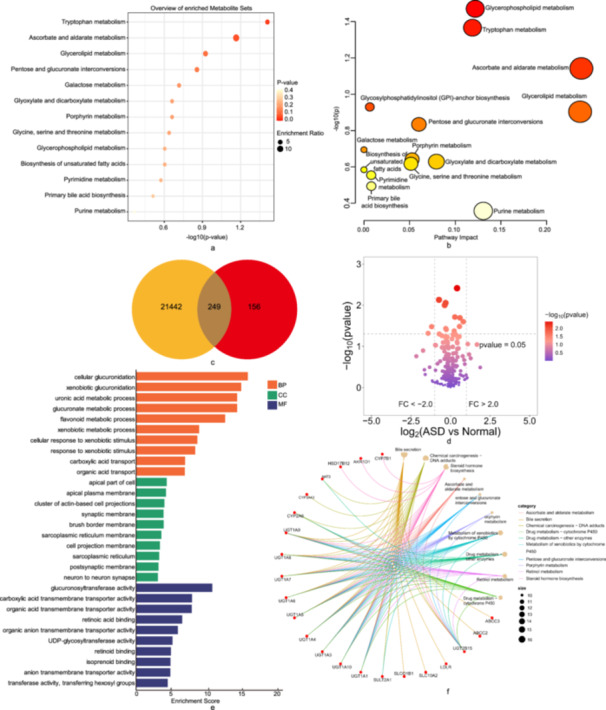
Potential metabolic pathways and network pharmacology analysis associated with ASD. (a, b) The metabolic pathways of blood metabolites (c) Venn plot of potential targets of blood metabolites‐related ASD (d) The volcano plot of the related genes (e) Enriched GO terms for BP analysis, CC analysis and MF analysis (f) KEGG pathway analysis. Abbreviations: ASD, autism spectrum disorder; BP, biological processes; CC, cellular components; GO, Gene Ontology; KEGG, Kyoto Encyclopedia of Genes and Genomes; MF, molecular functions.

We performed metabolite pathway analysis using all the metabolites discovered using the IVW approach (*p* < 0.05) [47]. Using forward MR analysis, we identified 13 potential metabolic pathways associated with ASD. The results showed that the tryptophan metabolism pathway (*p* = 0.0388) may participate in ASD genesis (Figure 8a,b). To delve deeper into the mechanisms by which blood metabolites influence ASD, we used network pharmacology approaches. Our findings, illustrated in a Venn plot, indicate that 249 genes were associated with both ASD and blood metabolites (Figure 8c, Table S3). Among these, 103 were upregulated, and 146 were downregulated (Figure 8d). For a more comprehensive understanding, we conducted GO functional analysis to assess the enrichment in BP, CC, and MF. The analysis revealed significant enrichment in several BP terms, namely “cellular glucuronidation,” “xenobiotic glucuronidation,” “uronic acid metabolic process,” “glucuronate metabolic process,” “flavonoid metabolic process,” “xenobiotic metabolic process,” “cellular response to xenobiotic stimulus,” “response to xenobiotic stimulus,” “carboxylic acid transport” and organic acid transport (Figure 8e). As for CC terms, the prominent enrichments were in the “apical part of cell,” “apical plasma membrane,” “cluster of actin‐based cell projections,” “synaptic membrane,” “brush border membrane,” “sarcoplasmic reticulum membrane,” “cell projection membrane,” “sarcoplasmic reticulum,” “postsynaptic membrane” and “neuron to neuron synapse” (Figure 8e). For MF, the principal enrichments were in functions such as “glucuronosyltransferase activity,” “carboxylic acid transmembrane transporter activity,” “organic acid transmembrane transporter activity,” “retinoic acid binding,” “organic anion transmembrane transporter activity,” “UDP‐glycosyltransferase activity,” “retinoid binding,” “isoprenoid binding,” “anion transmembrane transporter activity,” “transferase activity” and “transferring hexosyl groups” (Figure 8e). KEGG enrichment analysis revealed that related gene mainly involved in “Bile secretion,” “Chemical carcinogenesis ‐ DNA adducts,” “Steroid hormone biosynthesis,” “Ascorbate and aldarate metabolism,” “Pentose and glucuronate interconversions,” “Porphyrin metabolism,” “Metabolism of xenobiotics by cytochrome P450,” “Drug metabolism ‐ other enzymes,” “Retinol metabolism,” and “Drug metabolism ‐ cytochrome P450” (Figure 8f).

## 4 Discussion

Through extensive MR analysis, this study robustly identified two key blood metabolites, C12:1‐DC and methionine sulfone, along with two critical ratios, cysteine to alanine and proline to glutamate, that exhibited a causal relationship with ASD. IVW analysis, supplemented by a series of assessments, provides preliminary evidence to support the hypothesis that these metabolites and their ratios play a significant role in the progression of ASD. These findings provide novel insights into the metabolic pathways involved in ASD etiology. The identification of these metabolites and their ratios underscores the complexity of biological underpinnings of ASD. Their roles in the central nervous system have also been reported.

### 4.1 C12:1‐DC and ASD

In our study, we found that C12:1‐DC levels are higher in ASD. C12:1‐DC, also known as traumatic acid, is associated with several metabolic pathways [48]. This increase may be associated with alterations in fatty acid metabolism [48], which is known to be disrupted in ASD [49]. Studies have indicated that traumatic acid may hinder mitochondrial fatty acid β‐oxidation [50], which is essential for energy production in neurons [51]. In addition, while current research has yet to report on the effects of traumatic acid on ASD, several studies have shown that traumatic acid possesses antioxidant properties, regulates inflammatory responses, and promotes cell proliferation [48, 52, 53]. Traumatic acid has significant effects on promoting wound healing, reducing lipid accumulation, and alleviating traumatic brain injury (TBI) damage [48].

### 4.2 Methionine Sulfone and ASD

This study found that methionine sulfone elevation is a risk factor for ASD. It has been reported in the literature that levels of methionine sulfoxide in plasma and urine are significantly elevated in patients with ASD [54, 55]. Methionine sulfoxide is an oxidized form of methionine. Methionine is an essential amino acid involved in various metabolic processes in the body, including those affecting brain functions [56]. Under normal conditions methionine sulfone can be reduced back to methionine by the methionine sulfoxide reductase system to protect cells [57]. However, in ASD patients, elevated oxidative stress levels exacerbate the oxidation of methionine, while decreased reductase activity fails to effectively clear these oxidized products [58], resulting in the accumulation of methionine sulfoxide [59, 60, 61]. Additionally, the antioxidant defense system in ASD patients may be compromised, as evidenced by reduced levels of glutathione (GSH), which may also contribute to elevated methionine sulfoxide levels [61].

Abnormalities in the methionine metabolic pathway in ASD patients may lead to the accumulation of methionine sulfoxide, which in turn can impact neurodevelopment and function [62]. This accumulation may negatively affect neuronal function, particularly through its accumulation in proteins, potentially altering protein structure and function and impairing neural signal transmission and synaptic function [59]. Furthermore, the accumulation of methionine sulfoxide may disrupt neurotransmitter metabolism, further exacerbating ASD symptoms [63]. Third, methionine sulfone may play a role in managing oxidative stress in neuronal cells [64]. Fourth, as a metabolite of methionine, methionine sulfone may contribute to detoxification pathways in the brain [65]. Effective detoxification is crucial for maintaining neuronal health and function, which could be particularly important in neurodevelopmental disorders like ASD [66]. Fifth, methionine and its metabolites are key components in the methylation pathway [67], which is essential for numerous cellular processes, including DNA and protein synthesis [68]. Abnormalities in these pathways have been linked to various neurological conditions, suggesting that methionine sulfone may affect these metabolic pathways.

### 4.3 Cysteine to Alanine Ratio and ASD

Our findings indicate a higher cysteine‐to‐alanine ratio is a risk factor for the occurrence of ASD, which contrasts with some previous studies that have reported decreased cysteine levels in ASD patients [69]. We believe this discrepancy may be due to various factors, including sample selection, measurement methods, and the heterogeneity of ASD. It is important to note that ASD is a complex neurodevelopmental disorder, and its biomarkers may vary due to subtypes and individual differences [70]. Furthermore, Cysteine is a component of glutathione, which is crucial for antioxidant defense. Reduced cysteine levels may be associated with increased oxidative stress in ASD. Our study may shed light on the complex interplay between oxidative stress and amino acid metabolism in ASD, potentially influencing the cysteine‐to‐alanine ratio [70]. In summary, our research findings offer a distinct perspective on the cysteine‐to‐alanine ratio in ASD. This discrepancy may reflect the complexity of ASD pathophysiology and the heterogeneity observed across different studies.

Changing in amino acid proportions, may indicate underlying biochemical abnormalities associated with neurodevelopmental and neurodegenerative conditions [71, 72]. A high cysteine to alanine ratio could suggest an enhanced ability to synthesize glutathione, potentially counteracting oxidative stress, which is implicated in the pathophysiology of ASD [73]. Additionally, cysteine can affect excitatory neurotransmitter systems through its involvement in taurine and glutathione synthesis [74], potentially influencing neuronal excitability and signaling [75, 76, 77]. Glutamate is the main excitatory neurotransmitter in the CNS [78]. Therefore, we believe that further research is warranted to explore the underlying mechanisms of these ratio changes and to validate these differences in a larger sample size.

### 4.4 Proline to Glutamate Ratio and ASD

This study found that a decreased proline to glutamate ratio is a risk factor for the occurrence of ASD. Multiple studies have discovered that individuals with ASD have significantly lower levels of proline in their serum or urine [79, 80, 81]. Conversely, ASD patients exhibit significantly elevated levels of glutamate in their serum or brain [82, 83, 84]. Additionally, the ratio of proline to glutamate in the serum or urine of ASD patients is significantly altered [79, 80]. ASD patients have reduced levels of proline and increased levels of glutamate in their urine, leading to a significant decrease in the proline to glutamate ratio [79].

**Figure 9:**
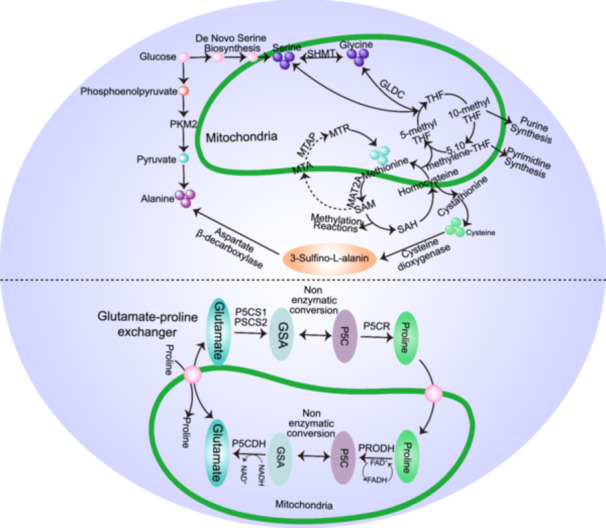
Metabolic pathway of potential blood metabolites. Abbreviations: GLDC, glycine dehydrogenase complex; GSA, gamma‐glutamylcysteine synthetase; 5‐methyl THF, 5‐methyltetrahydrofolate; 10‐methyl THF, 10‐methyltetrahydrofolate; MTA, methionine adenosyltransferase; MTR, methionine synthase; MTRR, methionine synthase reductase; P5C, pyrroline‐5‐carboxylate; P5CDH, pyrroline‐5‐carboxylate dehydrogenase; P5CR, pyrroline‐5‐carboxylate reductase; PKM2, pyruvate Kinase M 2; PRODH, proline dehydrogenase; PSCS1, proline synthase complex subunit 1; PSCS2, proline synthase complex subunit 2; SAH, S‐adenosylhomocysteine; SAM, S‐adenosylmethionine; SHMT, serine hydroxymethyltransferase; THF, tetrahydrofolate.

An altered proline to glutamate ratio may indicate changes in the excitatory synaptic activity or glutamate metabolism. Furthermore, proline modulates glutamate receptors and may affect synaptic plasticity [85]. Abnormal proline levels have been associated with neuropsychiatric disorders [86, 87], suggesting that this ratio may have implications for synaptic function and cognitive outcomes in ASD. These ratios might not only reflect current metabolic states but could also serve as biomarkers for the diagnosis or progression of neurological conditions. Understanding these ratios in the context of ASD could lead to the development of biomarkers that can help in early diagnosis or assessment of treatment efficacy. Modifying these ratios through diet or medication may offer new avenues for therapy aimed at restoring metabolic balance and possibly improving clinical outcomes. Researchers commonly employ biochemical and molecular biological methods to examine these ratios in both clinical and experimental contexts. Data analysis aids in decoding the intricate interplay between metabolic processes and brain functions in neurodevelopmental and neurodegenerative disorders (Figure 9).

### 4.5 Metabolic Pathway and ASD

In this study, metabolic pathway analysis identified tryptophan metabolism as the crucial important pathway in the effect of blood metabolites on ASD the same as the report. Tryptophan metabolism plays a critical role in the hippocampus [88]. This crucial metabolic pathway influences several key aspects of neurological function through its products, serotonin and kynurenine [89]. Tryptophan is a precursor of serotonin [90], a neurotransmitter heavily involved in mood regulation, social behavior, and cognitive functions [91, 92]. Serotonin imbalances are often found in individuals with ASD [93], suggesting that disturbances in tryptophan metabolism could contribute to the symptomatology observed in these disorders. Tryptophan is also metabolized along the kynurenine pathway [94, 95], which produces several neuroactive metabolites [96]. These metabolites can affect neuroplasticity, neuroinflammation, and excitotoxicity [97], and have been implicated in the neuropathology of ASD [98, 99]. Anomalies in this pathway may lead to elevated levels of neurotoxic compounds or reduced neuroprotective compounds [100], influencing the severity and presentation of ASD symptoms. Tryptophan and its metabolites influence several neural circuits and systems integral to cognitive and emotional processing. An imbalance in the level of these metabolites can disrupt these processes. Understanding the role of tryptophan metabolism in ASD opens potential avenues for therapeutic interventions. Modulation of this pathway through dietary supplementation, enzyme inhibitors, or receptor modulators could provide new strategies for managing symptoms associated with ASD.

### 4.6 Network Pharmacology and ASD

Moreover, we combined network pharmacology with MR to provide a robust framework for understanding the genetic and metabolic underpinnings of ASD, which facilitates the elucidation of complex bio‐networks and aids in distinguishing between correlational and causal relationships in the pathology. GO analysis revealed that BPs, CCs, and MFs were enriched among these related genes, providing a deeper insight into how these genetic changes may influence cellular behavior and metabolic processes. BPs such as “xenobiotic metabolic process” and “cellular response to xenobiotic stimulus” suggest heightened cellular interactions with foreign compounds [101], possibly reflecting environmental interactions in ASD etiology. CC results reveal critical involvement of cellular structures like “synaptic membrane” and “neuron to neuron synapse” pertinent to neurodevelopment and synaptic plasticity known to be altered in ASD [102]. MF enrichment emphasizes activities such as “transmembrane transporter activity,” suggesting alterations in cellular transport mechanisms that could impact ion homeostasis and neurotransmitter levels, thereby influencing neuronal function [103]. And the KEGG enrichment analysis pinpointed the significant involvement of specific genes in various metabolic and biosynthetic pathways, notably in “Bile secretion,” “Chemical carcinogenesis‐DNA adducts,” and several other key metabolic processes. Hence, network pharmacology is pivotal for enriching the scope and depth of MR studies, providing a robust framework for understanding complex disease mechanisms, and improving the reliability of epidemiological findings.

### 4.7 Limitations

However, this study has some limitations. The MR approach, which is powerful in inferring causality, relies on the assumption that the genetic variants used as instruments are not associated with confounding factors [104]. Although stringent criteria have been applied to select these variants, the possibility of pleiotropy could not be entirely excluded [105]. Additionally, the generalizability of these findings may be limited by the population characteristics of the study sample, and further validation in diverse cohorts is necessary. Future research should focus on elucidating the biological mechanisms by which these metabolites and their ratios influence ASD development. Experimental studies in cellular and animal models as well as targeted metabolomic analyses in humans could provide valuable insights. Furthermore, exploring the potential of these metabolites and their ratios as biomarkers for ASD risk or as targets for therapeutic intervention could have significant clinical implications.

## 5 Conclusions

In summary, this study may contribute to personalized interpretation, highlight biological disparities in disease states, and identify potential molecules for future mechanistic investigations. A comparison of our findings with existing literature reveals both consistencies and novel insights. Previous studies have implicated lipid and amino acid metabolism in ASD; however, the specific metabolites and ratios identified in this study provide new targets for further investigation. Our study highlights the potential involvement of specific metabolites and their ratios in ASD risk, offering new avenues for understanding the metabolic underpinnings of this complex disorder. While the direct effects of metabolites on ASD are not fully understood, their role in metabolism and potential impact on brain development warrants further investigation. These findings underscore the importance of integrating genetic and metabolomic data to uncover the biological basis of neurodevelopmental disorders and to pave the way for novel diagnostic and therapeutic strategies.

## Author Contributions

**Wenhua Li:** conceptualization, methodology, writing–original draft, writing–review and editing, project administration, data curation, software, and supervision. **Suya Ma:** formal analysis. **Yunong Tian:** writing–original draft, and visualization. **All authors:** read and approved the final version of the manuscript.

## Ethics Statement

The authors have nothing to report.

## Consent

The authors have nothing to report.

## Conflicts of Interest

The authors declare no conflicts of interest.

## Transparency Statement

The lead author Wenhua Li affirms that this manuscript is an honest, accurate, and transparent account of the study being reported; that no important aspects of the study have been omitted; and that any discrepancies from the study as planned (and, if relevant, registered) have been explained.

## Supporting information

Supporting Fig. 1: Forest plot for the causality of blood metabolites on ASD derived from MR Egger analysis.

Supporting Fig. 2: Forest plot for the causality of blood metabolites on ASD derived from weighted median analysis.

Supporting Fig. 3: Forest plot for the causality of blood metabolites on ASD derived from simple mode analysis.

Supporting Fig. 4: Forest plot for the causality of blood metabolites on ASD derived from weighted mode analysis.

Supporting Fig. 5: Leave‐One‐Out plots illustrating the causal associations between blood metabolites and ASD.

Supporting Fig. 6: Funnel plots to visualize the overall heterogeneity of MR estimates for the causal associations between blood metabolites and ASD.

Supporting Fig. 7: Replication analysis of the causal relationship between blood metabolites and ASD.

Supporting Fig. 8. Reserve analysis of the final identified blood metabolites.

Supporting information.

Supporting information.

Supporting information.

## Data Availability

The data that support the findings of this study are available from the corresponding author upon reasonable request. Wenhua Li had had full access to all of the data in this study and takes complete responsibility for the integrity of the data and the accuracy of the data analysis.
