# Supplementary figures and images for: Exploring the Association Between Human Blood Metabolites and Autism Spectrum Disorder Risk: A Bidirectional Mendelian Randomization Study
Source: Health Sci Rep. 2025 Mar 3;8(3):e70528. doi: 10.1002/hsr2.70528 (PMC11875788; doi:10.1002/hsr2.70528)

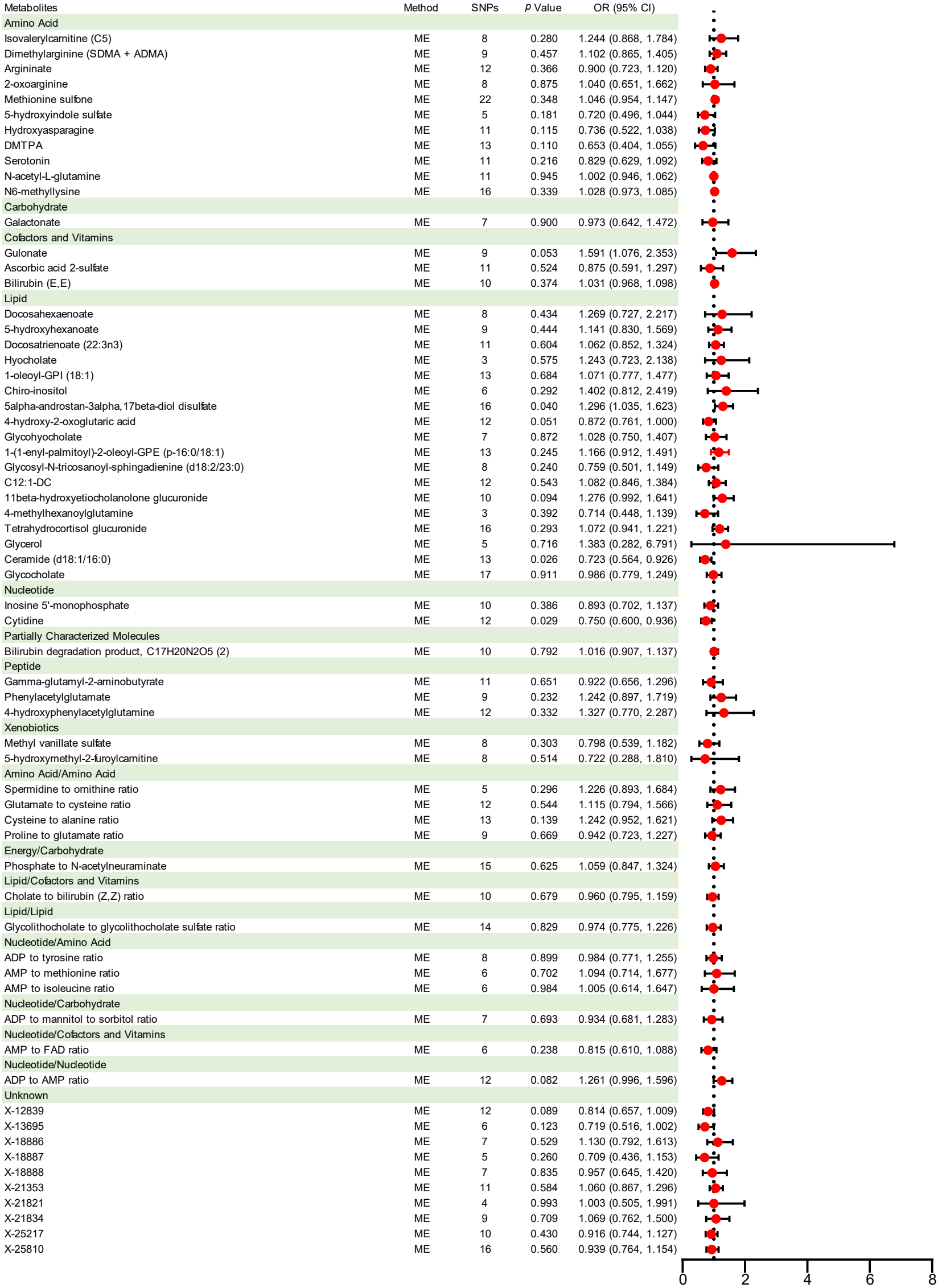

Supplement: Supplementary file 1 — Supporting Fig. 1: Forest plot for the causality of blood metabolites on ASD derived from MR Egger analysis. [file HSR2-8-e70528-s011.pdf]

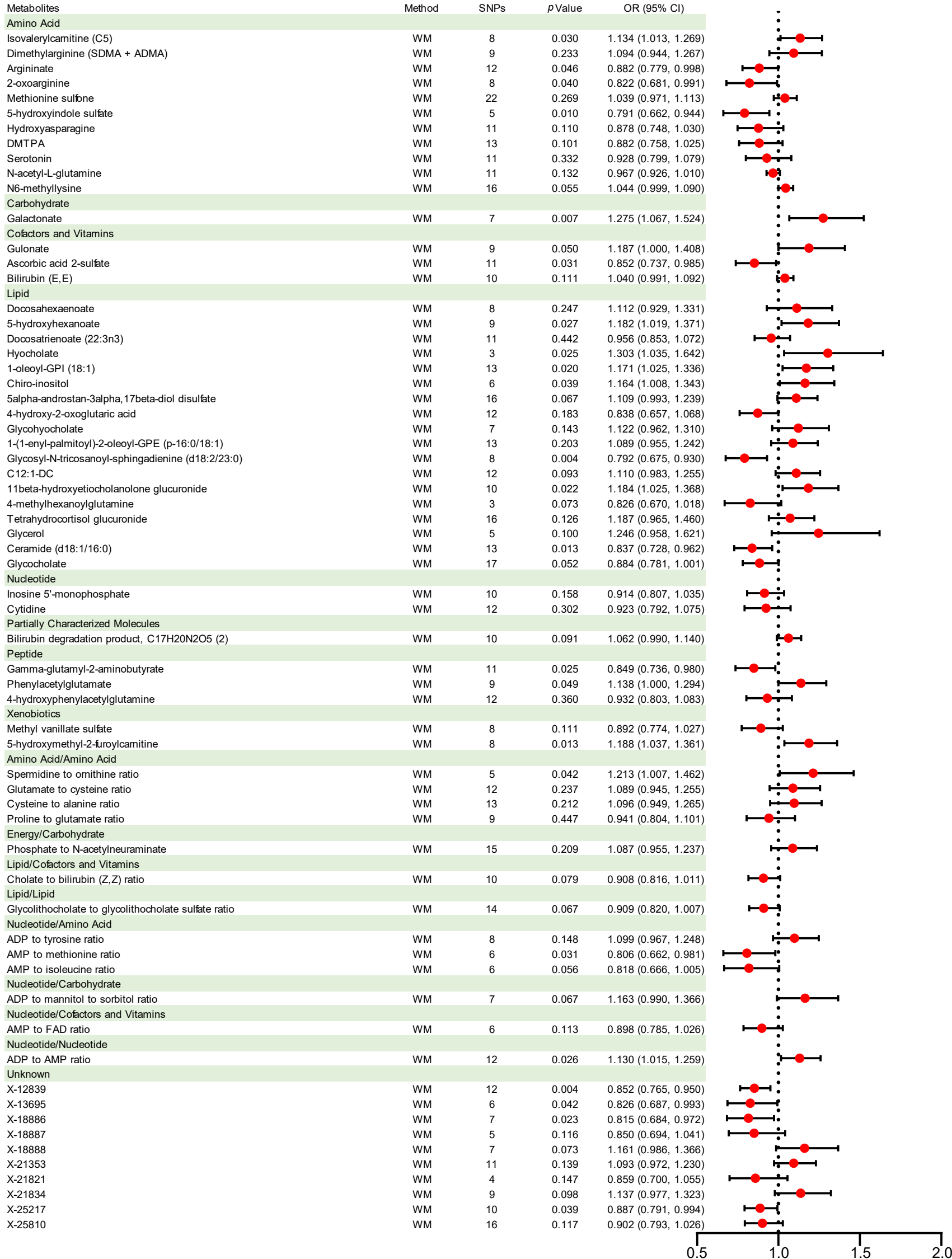

Supplement: Supplementary file 2 — Supporting Fig. 2: Forest plot for the causality of blood metabolites on ASD derived from weighted median analysis. [file HSR2-8-e70528-s010.pdf]

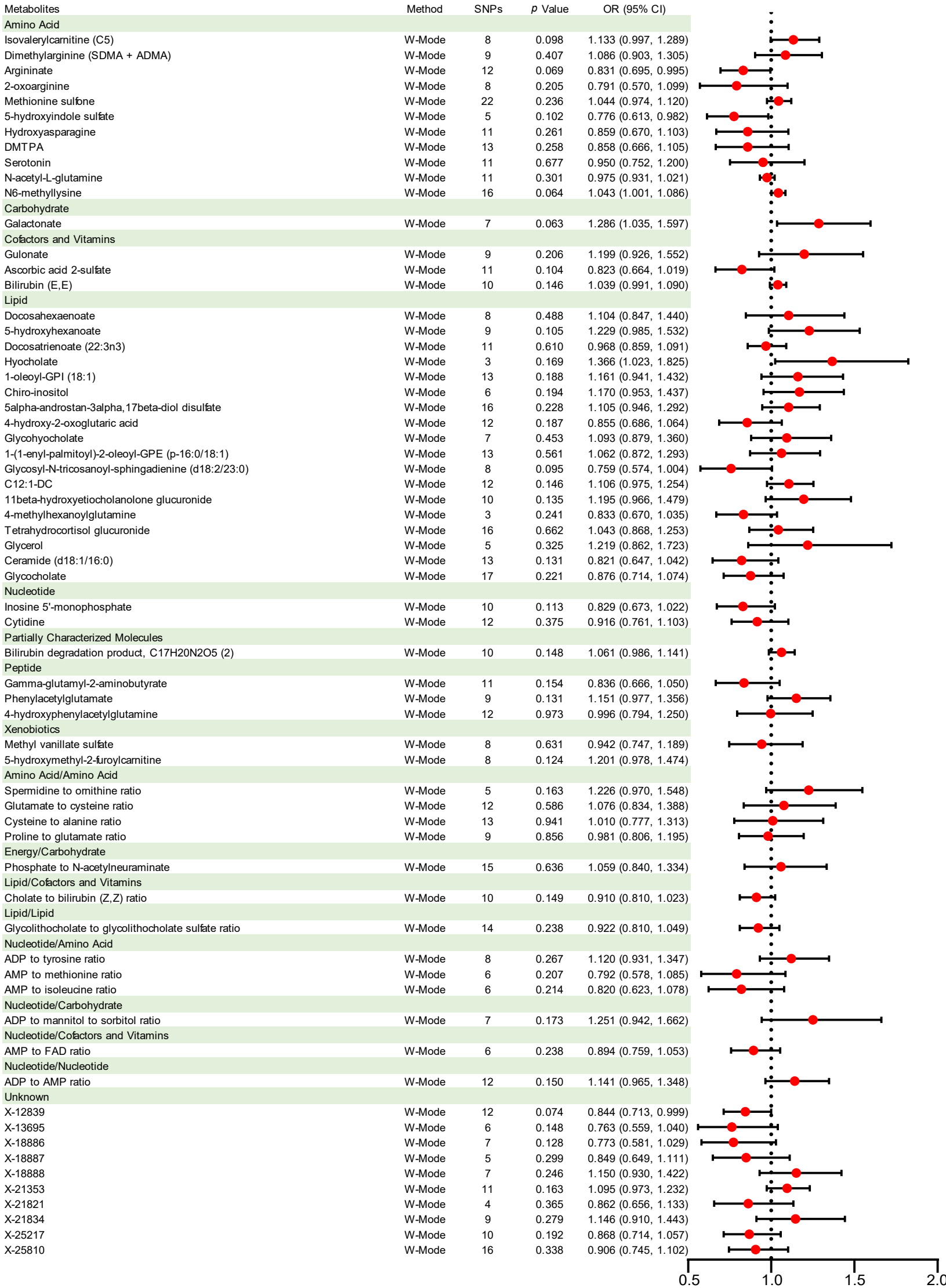

Supplement: Supplementary file 4 — Supporting Fig. 4: Forest plot for the causality of blood metabolites on ASD derived from weighted mode analysis. [file HSR2-8-e70528-s006.pdf]

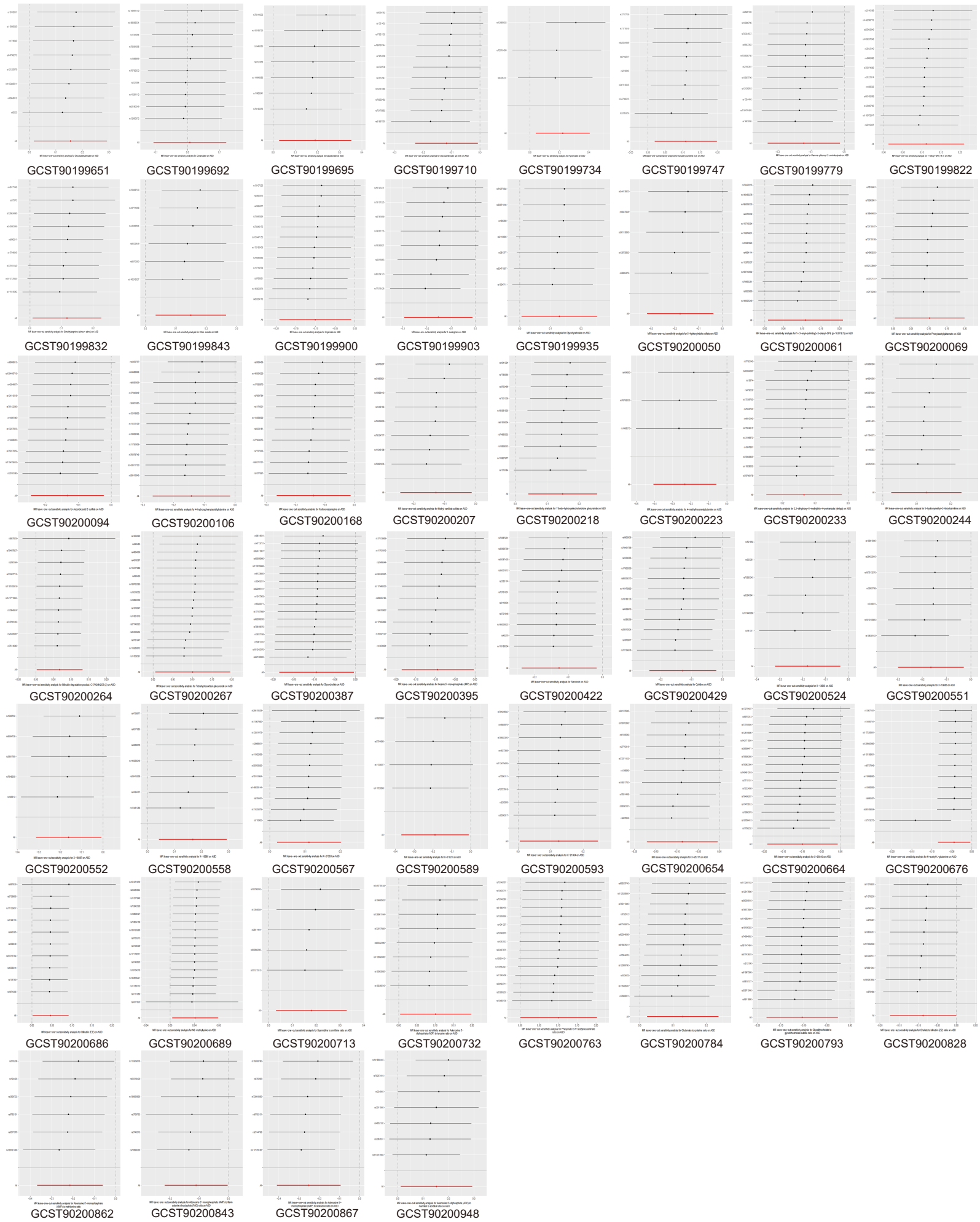

Supplement: Supplementary file 5 — Supporting Fig. 5: Leave‐One‐Out plots illustrating the causal associations between blood metabolites and ASD. [file HSR2-8-e70528-s005.pdf]

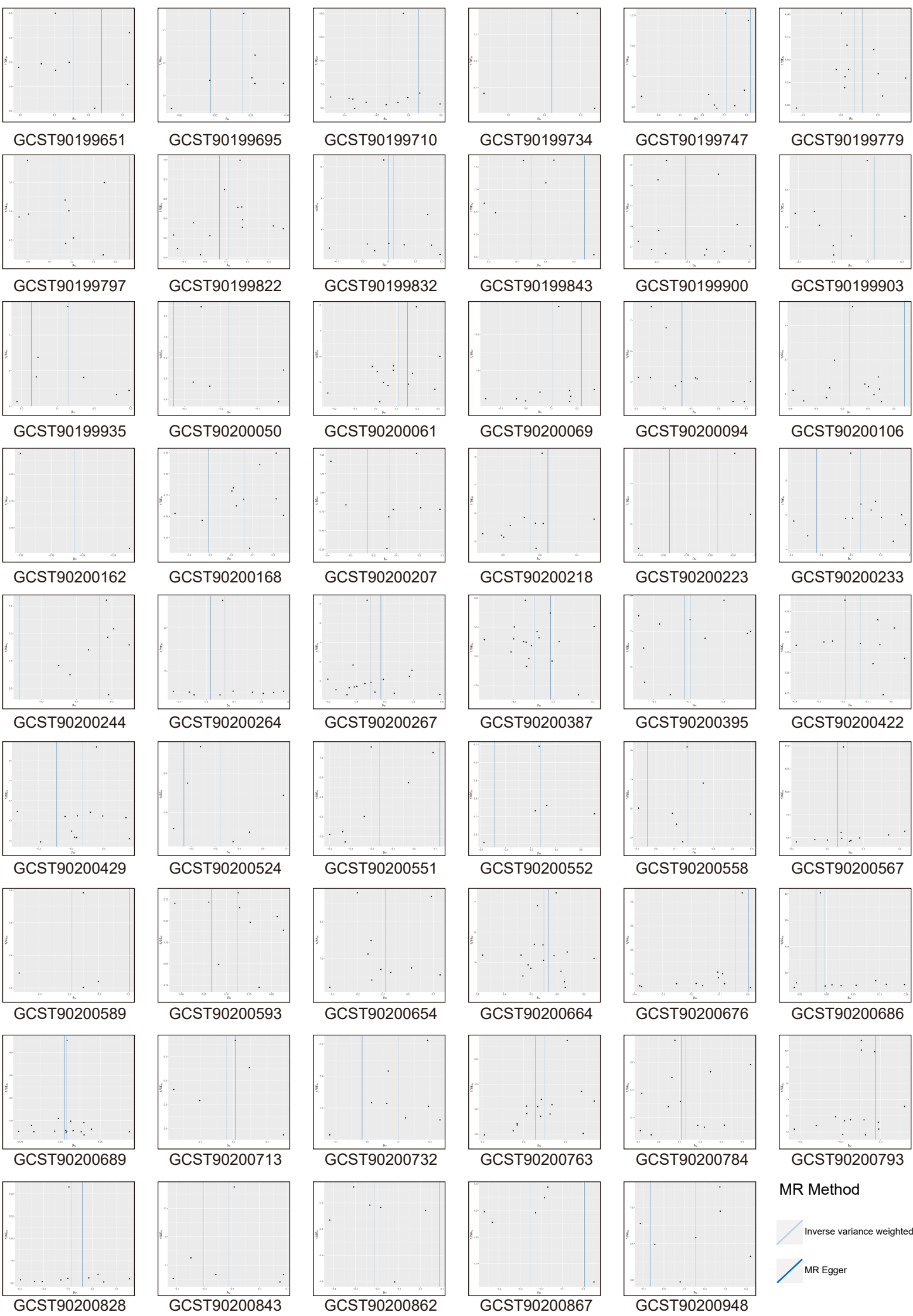

Supplement: Supplementary file 6 — Supporting Fig. 6: Funnel plots to visualize the overall heterogeneity of MR estimates for the causal associations between blood metabolites and ASD. [file HSR2-8-e70528-s007.pdf]
