# Supplementary material for: Exploring the Association Between Human Blood Metabolites and Autism Spectrum Disorder Risk: A Bidirectional Mendelian Randomization Study
Source: Health Sci Rep. 2025 Mar 3;8(3):e70528. doi: 10.1002/hsr2.70528 (PMC11875788; doi:10.1002/hsr2.70528)

Metabolites	Method	SNPs	*p* Value	OR (95% CI)
Amino Acid				
Isovalerylcarnitine (C5)	SM	8	0.345	1.087 (0.925, 1.276)
Dimethylarginine (SDMA + ADMA)	SM	9	0.492	1.086 (0.868, 1.358)
Argininate	SM	12	0.093	0.828 (0.677, 1.012)
2-oxoarginine	SM	8	0.188	0.795 (0.584, 1.082)
Methionine sulfone	SM	22	0.364	1.067 (0.930, 1.225)
5-hydroxyindole sulfate	SM	5	0.128	0.777 (0.600, 1.006)
Hydroxyasparagine	SM	11	0.305	0.859 (0.653, 1.131)
DMTPA	SM	13	0.246	0.854 (0.662, 1.101)
Serotonin	SM	11	0.677	0.950 (0.752, 1.200)
N-acetyl-L-glutamine	SM	11	0.205	0.904 (0.781, 1.046)
N6-methyllysine	SM	16	0.372	1.060 (0.936, 1.201)
Carbohydrate				
Galactonate	SM	7	0.080	1.299 (1.018, 1.659)
Cofactors and Vitamins				
Gulonate	SM	9	0.224	1.209 (0.911, 1.604)
Ascorbic acid 2-sulfate	SM	11	0.169	0.840 (0.666, 1.058)
Bilirubin (E,E)	SM	10	0.469	1.057 (0.916, 1.220)
Lipid				
Docosahexaenoate	SM	8	0.476	1.109 (0.847, 1.453)
5-hydroxyhexanoate	SM	9	0.123	1.218 (0.973, 1.523)
Docosatrienoate (22:3n3)	SM	11	0.720	0.950 (0.724, 1.247)
Hyocholate	SM	3	0.150	1.371 (1.045, 1.798)
1-oleoyl-GPI (18:1)	SM	13	0.189	1.161 (0.941, 1.433)
Chiro-inositol	SM	6	0.236	1.172 (0.930, 1.477)
5alpha-androstan-3alpha,17beta-diol disulfate	SM	16	0.308	1.105 (0.918, 1.331)
4-hydroxy-2-oxoglutaric acid	SM	12	0.194	0.847 (0.669, 1.072)
Glycohyocholate	SM	7	0.602	1.065 (0.850, 1.335)
1-(1-enyl-palmitoyl)-2-oleoyl-GPE (p-16:0/18:1)	SM	13	0.644	1.054 (0.847, 1.312)
Glycosyl-N-tricosanoyl-sphingadienine (d18:2/23:0)	SM	8	0.087	0.754 (0.571, 0.996)
C12:1-DC	SM	12	0.109	1.217 (0.976, 1.517)
11beta-hydroxyetiocholanolone glucuronide	SM	10	0.179	1.183 (0.944, 1.483)
4-methylhexanoylglutamine	SM	3	0.305	0.837 (0.648, 1.080)
Tetrahydrocortisol glucuronide	SM	16	0.892	1.016 (0.814, 1.267)
Glycerol	SM	5	0.291	1.222 (0.885, 1.688)
Ceramide (d18:1/16:0)	SM	13	0.143	0.821 (0.642, 1.051)
Glycocholate	SM	17	0.266	0.876 (0.699, 1.097)
Nucleotide				
Inosine 5'-monophosphate	SM	10	0.116	0.827 (0.667, 1.024)
Cytidine	SM	12	0.218	0.852 (0.671, 1.083)
Partially Characterized Molecules				
Bilirubin degradation product, C17H20N2O5 (2)	SM	10	0.315	1.096 (0.926, 1.296)
Peptide				
Gamma-glutamyl-2-aminobutyrate	SM	11	0.125	0.836 (0.677, 1.031)
Phenylacetylglutamate	SM	9	0.159	1.173 (0.959, 1.434)
4-hydroxyphenylacetylglutamine	SM	12	0.859	1.025 (0.786, 1.336)
Xenobiotics				
Methyl vanillate sulfate	SM	8	0.565	0.929 (0.731, 1.181)
5-hydroxymethyl-2-furoylcarnitine	SM	8	0.128	1.203 (0.975, 1.483)
Amino Acid/Amino Acid				
Spermidine to ornithine ratio	SM	5	0.173	1.240 (0.961, 1.599)
Glutamate to cysteine ratio	SM	12	0.586	1.076 (0.834, 1.388)
Cysteine to alanine ratio	SM	13	0.954	1.008 (0.773, 1.315)
Proline to glutamate ratio	SM	9	0.775	0.963 (0.748, 1.238)
Energy/Carbohydrate				
Phosphate to N-acetylneuraminate	SM	15	0.709	1.047 (0.828, 1.323)
Lipid/Cofactors and Vitamins				
Cholate to bilirubin (Z,Z) ratio	SM	10	0.338	0.910 (0.759, 1.092)
Lipid/Lipid				
Glycolithocholate to glycolithocholate sulfate ratio	SM	14	0.302	0.915 (0.778, 1.076)
Nucleotide/Amino Acid				
ADP to tyrosine ratio	SM	8	0.254	1.132 (0.931, 1.376)
AMP to methionine ratio	SM	6	0.204	0.809 (0.609, 1.075)
AMP to isoleucine ratio	SM	6	0.243	0.814 (0.600, 1.104)
Nucleotide/Carbohydrate				
ADP to mannitol to sorbitol ratio	SM	7	0.178	1.245 (0.939, 1.651)
Nucleotide/Cofactors and Vitamins				
AMP to FAD ratio	SM	6	0.138	0.827 (0.670, 1.021)
Nucleotide/Nucleotide				
ADP to AMP ratio	SM	12	0.155	1.146 (0.962, 1.366)
Unknown				
X-12839	SM	12	0.071	0.839 (0.706, 0.996)
X-13695	SM	6	0.132	0.761 (0.565, 1.025)
X-18886	SM	7	0.087	0.739 (0.553, 0.988)
X-18887	SM	5	0.298	0.849 (0.650, 1.110)
X-18888	SM	7	0.309	1.134 (0.909, 1.415)
X-21353	SM	11	0.370	1.102 (0.900, 1.348)
X-21821	SM	4	0.376	0.864 (0.654, 1.140)
X-21834	SM	9	0.257	1.152 (0.918, 1.445)
X-25217	SM	10	0.199	0.870 (0.714, 1.060)
X-25810	SM	16	0.223	0.884 (0.731, 1.069)

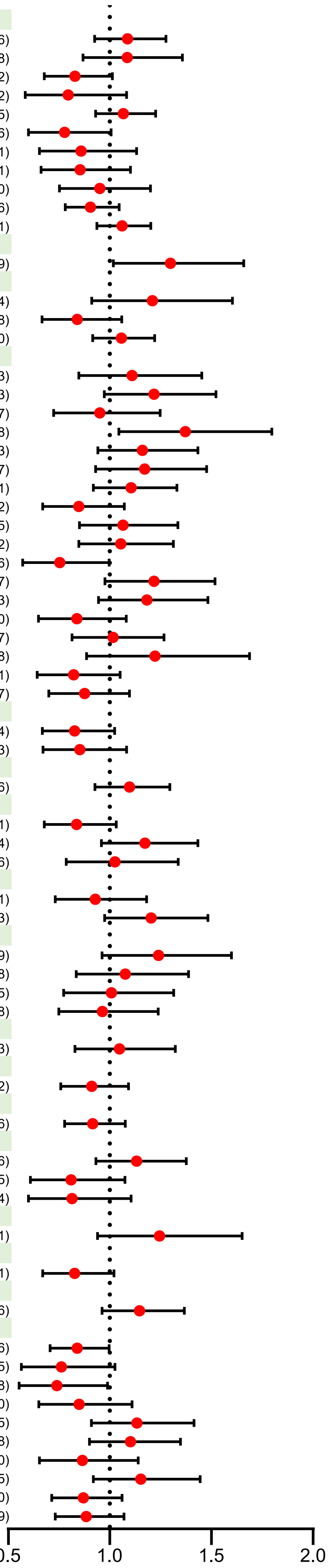

Supplement: Supplementary file 3 — Supporting Fig. 3: Forest plot for the causality of blood metabolites on ASD derived from simple mode analysis. [file HSR2-8-e70528-s003.pdf]
