# Supplementary material for: Exploring the Association Between Human Blood Metabolites and Autism Spectrum Disorder Risk: A Bidirectional Mendelian Randomization Study
Source: Health Sci Rep. 2025 Mar 3;8(3):e70528. doi: 10.1002/hsr2.70528 (PMC11875788; doi:10.1002/hsr2.70528)
Supplement: Supplementary file 7 — Supporting Fig. 7: Replication analysis of the causal relationship between blood metabolites and ASD. [file HSR2-8-e70528-s009.pdf]

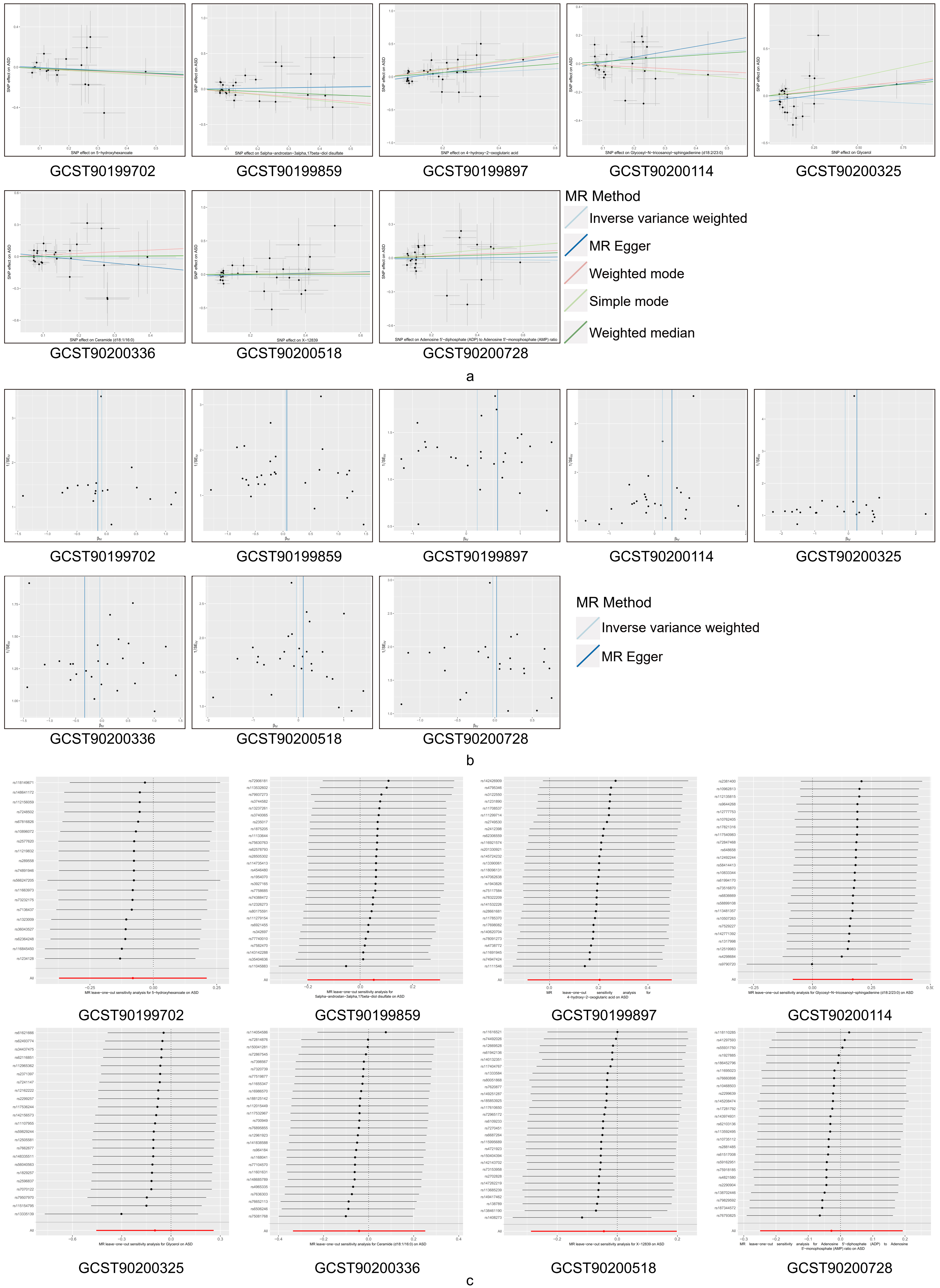

SNP effect on 5-hydroxyhexanoate
SNP effect on 5alpha-androstan-3alpha,17beta-diol disulfate
SNP effect on 4-hydroxy-2-oxoglutaric acid
SNP effect on Glycosyl-N-tricosanoyl-sphingadienine (d18:2/23:0)
SNP effect on Glycerol
SNP effect on Ceramide (d18:1/16:0)
SNP effect on X-12839
SNP effect on Adenosine 5'-diphosphate (ADP) to Adenosine 5'-monophosphate (AMP) ratio
SNP effect on ASD
GCST90199702
GCST90199859
GCST90199897
GCST90200114
GCST90200325
GCST90200336
GCST90200518
GCST90200728
MR Method
Inverse variance weighted
MR Egger
Weighted mode
Simple mode
Weighted median
a
GCST90199702
GCST90199859
GCST90199897
GCST90200114
GCST90200325
GCST90200336
GCST90200518
GCST90200728
MR Method
Inverse variance weighted
MR Egger
b
MR leave-one-out sensitivity analysis for 5-hydroxyhexanoate on ASD
MR leave-one-out sensitivity analysis for 5alpha-androstan-3alpha,17beta-diol disulfate on ASD
MR leave-one-out sensitivity analysis for 4-hydroxy-2-oxoglutaric acid on ASD
MR leave-one-out sensitivity analysis for Glycosyl-N-tricosanoyl-sphingadienine (d18:2/23:0) on ASD
MR leave-one-out sensitivity analysis for Glycerol on ASD
MR leave-one-out sensitivity analysis for Ceramide (d18:1/16:0) on ASD
MR leave-one-out sensitivity analysis for X-12839 on ASD
MR leave-one-out sensitivity analysis for Adenosine 5'-diphosphate (ADP) to Adenosine 5'-monophosphate (AMP) ratio on ASD
GCST90199702
GCST90199859
GCST90199897
GCST90200114
GCST90200325
GCST90200336
GCST90200518
GCST90200728
c
