# Supplementary material for: Exploring the Association Between Human Blood Metabolites and Autism Spectrum Disorder Risk: A Bidirectional Mendelian Randomization Study
Source: Health Sci Rep. 2025 Mar 3;8(3):e70528. doi: 10.1002/hsr2.70528 (PMC11875788; doi:10.1002/hsr2.70528)
Supplement: Supplementary file 8 — Supporting Fig. 8. Reserve analysis of the final identified blood metabolites. [file HSR2-8-e70528-s004.pdf]

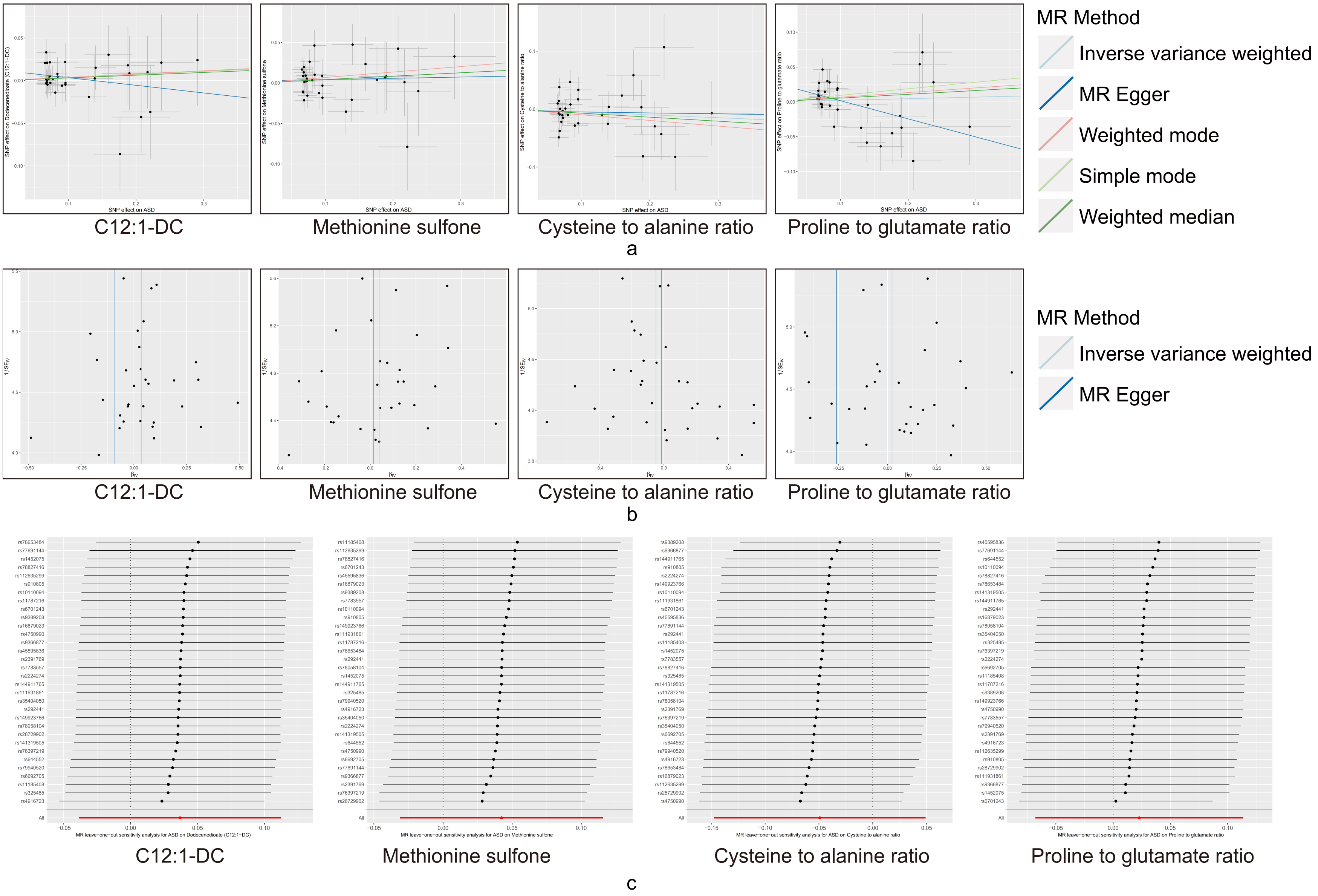
MR Method
Inverse variance weighted
MR Egger
Weighted mode
Simple mode
Weighted median
C12:1-DC
Methionine sulfone
Cysteine to alanine ratio
Proline to glutamate ratio
a
MR Method
Inverse variance weighted
MR Egger
C12:1-DC
Methionine sulfone
Cysteine to alanine ratio
Proline to glutamate ratio
b
C12:1-DC
Methionine sulfone
Cysteine to alanine ratio
Proline to glutamate ratio
c
