# Supplementary material for: Exploring the Association Between Human Blood Metabolites and Autism Spectrum Disorder Risk: A Bidirectional Mendelian Randomization Study
Source: Health Sci Rep. 2025 Mar 3;8(3):e70528. doi: 10.1002/hsr2.70528 (PMC11875788; doi:10.1002/hsr2.70528)
Supplement: Supplementary file 9 — Supporting information. [file HSR2-8-e70528-s001.docx]

**Table S1** Results of the query in Phenoscanner for positive metabolites after replication analysis

| Metabolites | Method | Before confounding analysis | | | After confounding analysis | | |
| --- | --- | --- | --- | --- | --- | --- | --- |
|  |  | SNPs | *p* Value | OR (95% CI) | SNPs | *p* Value | OR (95% CI) |
| Dodecenedioate (C12:1-DC) | IVW | 12 | 0.003 | 1.146 (1.046, 1.255) | 12 | 0.003 | 1.146 (1.046, 1.255) |
| Methionine sulfone | IVW | 22 | 0.031 | 1.059 (1.005, 1.116) | 22 | 0.031 | 1.059 (1.005, 1.116) |
| Cysteine to alanine ratio | IVW | 13 | 0.005 | 1.164 (1.048, 1.295) | 13 | 0.005 | 1.164 (1.048, 1.295) |
| Proline to glutamate ratio | IVW | 9 | 0.035 | 0.883 (0.786, 0.991) | 9 | 0.035 | 0.883 (0.786, 0.991) |
