# Supplementary material for: Exploring the Association Between Human Blood Metabolites and Autism Spectrum Disorder Risk: A Bidirectional Mendelian Randomization Study
Source: Health Sci Rep. 2025 Mar 3;8(3):e70528. doi: 10.1002/hsr2.70528 (PMC11875788; doi:10.1002/hsr2.70528)
Supplement: Supplementary file 10 — Supporting information. [file HSR2-8-e70528-s008.docx]

**Table S2** Confounders identified by Phenoscanner

| Metabolites | Method | Before confounding analysis | | | After confounding analysis | | | SNP | Trait |
| --- | --- | --- | --- | --- | --- | --- | --- | --- | --- |
|  |  | SNPs | *p* Value | OR (95% CI) | SNPs | *p* Value | OR (95% CI) |  |  |
| Galactonate | IVW | 7 | 0.022 | 1.209 (1.028, 1.423) | 6 | 0.074 | 1.205 (0.982, 1.478) | rs1446585 | Education, BMI, GM |
| Argininate | IVW | 12 | 0.031 | 0.904 (0.826, 0.991) | 11 | 0.094 | 0.919 (0.833, 1.014) | rs13107325 | Education, BMI, Intelligence |
| N-acetyl-L-glutamine | IVW | 11 | 0.030 | 0.957 (0.919, 0.996) | 10 | 0.002 | 0.868 (0.794, 0.949) | rs7573275 | Education |
| N6-methyllysine | IVW | 16 | 0.033 | 1.042 (1.003, 1.082) | 15 | 0.040 | 1.041 (1.002, 1.081) | rs10164318 | Education |
| AMP to FAD ratio | IVW | 6 | 0.044 | 0.895 (0.804, 0.997) | 5 | 0.121 | 0.883 (0.754, 1.033) | rs2706762 | Education, Intelligence |
| DMTPA | IVW | 13 | 0.022 | 0.875 (0.780, 0.981) | 12 | 0.020 | 0.865 (0.766, 0.977) | rs1047891 | Dietary factors, Body weight |
| X-12839 | IVW | 12 | 0.002 | 0.881 (0.814, 0.955) | 11 | 0.001 | 0.866 (0.797, 0.941) | rs138789 | GM |
| X-25810 | IVW | 16 | 0.034 | 0.905 (0.825, 0.993) | 15 | 0.149 | 0.932 (0.848, 1.025) | rs17279437 | BMI |
