# Supplementary material for: Exploring the Association Between Human Blood Metabolites and Autism Spectrum Disorder Risk: A Bidirectional Mendelian Randomization Study
Source: Health Sci Rep. 2025 Mar 3;8(3):e70528. doi: 10.1002/hsr2.70528 (PMC11875788; doi:10.1002/hsr2.70528)
Supplement: Supplementary file 11 — Supporting information. [file HSR2-8-e70528-s002.docx]

# Table S3 659 SNPs in blood metabolites associated ASD

| **SNP** | **Beta** | **se** | **P** | **Gene** |
| --- | --- | --- | --- | --- |
| rs10022462 | 0.067547 | 0.0146699 | 4.14E-06 | PPM1K-DT |
| rs1007161 | 0.123625 | 0.0261218 | 2.22E-06 | SLC7A9 |
| rs10098706 | 0.092922 | 0.0196733 | 2.32E-06 | / |
| rs1012976 | -0.22996 | 0.047234 | 1.12E-06 | VPS41 |
| rs1016647 | -0.09361 | 0.015879 | 3.74E-09 | CDC23 |
| rs1017363 | 0.07348 | 0.0160133 | 4.46E-06 | GABRG3, LOC124903449 |
| rs1018091 | 0.101809 | 0.0218657 | 3.22E-06 | FREM3 |
| rs10199322 | -0.07842 | 0.0147676 | 1.09E-07 | HECW2 |
| rs10201159 | 0.262208 | 0.0512087 | 3.05E-07 | / |
| rs10236510 | 0.256697 | 0.0561793 | 4.90E-06 | LINC02476 |
| rs1034711 | 0.217928 | 0.0475352 | 4.55E-06 | / |
| rs10400715 | -0.15539 | 0.0316191 | 8.90E-07 | TMEM63C |
| rs10422369 | 0.127946 | 0.0259802 | 8.45E-07 | / |
| rs10468503 | -0.08891 | 0.0180678 | 8.62E-07 | / |
| rs10489136 | 0.159531 | 0.0335054 | 1.92E-06 | / |
| rs10496399 | 0.083062 | 0.0181434 | 4.69E-06 | LINC02946, LOC107985929 |
| rs10503585 | 0.243472 | 0.0532321 | 4.79E-06 | LOC105379297 |
| rs10512976 | 0.085366 | 0.0176366 | 1.30E-06 | / |
| rs10762405 | -0.09457 | 0.0201894 | 2.81E-06 | / |
| rs1077697 | -0.08257 | 0.0176805 | 3.01E-06 | TTC7B |
| rs10808427 | -0.32628 | 0.065359 | 5.97E-07 | TMEM74 |
| rs10809267 | 0.102133 | 0.0223693 | 4.98E-06 | / |
| rs10816150 | -0.10327 | 0.0226087 | 4.93E-06 | PTPRD |
| rs10821702 | -0.08816 | 0.0179776 | 9.38E-07 | ANK3 |
| rs10833344 | 0.279215 | 0.0213512 | 4.44E-39 | / |
| rs10838163 | -0.17849 | 0.0389162 | 4.51E-06 | HSD17B12 |
| rs10838523 | -0.16651 | 0.0318086 | 1.65E-07 | / |
| rs1084950 | -0.16923 | 0.0369981 | 4.78E-06 | LOC105372130 |
| rs10892559 | 0.140451 | 0.0292856 | 1.62E-06 | POU2F3, LOC105369531 |
| rs10896190 | 0.426103 | 0.093 | 4.61E-06 | TBX10 |
| rs10918652 | 0.09228 | 0.0188518 | 9.83E-07 | LOC105371601 |
| rs10951036 | 0.100664 | 0.0180052 | 2.26E-08 | OSBPL3 |
| rs10957706 | -0.17024 | 0.0151609 | 2.93E-29 | JPH1 |
| rs11029652 | -0.17993 | 0.0151695 | 1.89E-32 | ANO3 |
| rs11045883 | 0.103185 | 0.0168317 | 8.76E-10 | SLCO1B1 |
| rs11052059 | -0.09174 | 0.019534 | 2.65E-06 | FGD4 |
| rs11061978 | 0.193812 | 0.0416167 | 3.21E-06 | SLC6A12 |
| rs11067600 | 0.126804 | 0.0267484 | 2.13E-06 | LOC105370003 |
| rs11076828 | 0.120653 | 0.0233346 | 2.33E-07 | CORO7, CORO7-PAM16 |
| rs11107955 | -0.16121 | 0.0345064 | 2.99E-06 | VEZT |
| rs1111546 | 0.085754 | 0.0179151 | 1.70E-06 | LINC02888 |
| rs11133665 | 0.220364 | 0.0477636 | 3.96E-06 | / |
| rs11161609 | -0.08306 | 0.0159675 | 1.97E-07 | DDAH1 |
| rs111837003 | -0.11439 | 0.0246897 | 3.60E-06 | BAZ1B |
| rs11189713 | 0.11632 | 0.0254208 | 4.74E-06 | HPSE2 |
| rs112015449 | -0.10067 | 0.0216653 | 3.38E-06 | KLHL40, LOC124906232 |
| rs1124174 | 0.082083 | 0.0159676 | 2.74E-07 | LOC124901785 |
| rs11245408 | 0.083949 | 0.017299 | 1.22E-06 | / |
| rs11246108 | -0.07702 | 0.0166028 | 3.51E-06 | / |
| rs112479426 | 0.258011 | 0.0467158 | 3.33E-08 | / |
| rs112520895 | 0.092518 | 0.0169223 | 4.57E-08 | / |
| rs112876557 | -0.08278 | 0.0169918 | 1.10E-06 | / |
| rs113286872 | 0.217786 | 0.0428227 | 3.66E-07 | VWA8-AS1 |
| rs1133607 | -0.09761 | 0.0211542 | 3.94E-06 | ACSM2A |
| rs113379968 | -0.08206 | 0.0178326 | 4.19E-06 | FAM13A |
| rs113592495 | 0.09422 | 0.019524 | 1.39E-06 | USP19 |
| rs113665619 | 0.13092 | 0.0246102 | 1.04E-07 | SORCS2 |
| rs113745941 | 0.100668 | 0.0220408 | 4.94E-06 | ARL15 |
| rs113874831 | 0.129058 | 0.0269972 | 1.75E-06 | / |
| rs113918643 | -0.08062 | 0.0175695 | 4.46E-06 | / |
| rs113935097 | -0.11524 | 0.0252109 | 4.85E-06 | PTPRA |
| rs113967277 | -0.25229 | 0.0465151 | 5.84E-08 | SLC22A9 |
| rs114558399 | 0.079884 | 0.0168616 | 2.16E-06 | / |
| rs114562444 | 0.087009 | 0.0148034 | 4.16E-09 | LOC102724340 |
| rs114735413 | -0.0708 | 0.0150933 | 2.72E-06 | LOC107985984, SMARCAL1-AS1 |
| rs115138061 | 0.091932 | 0.0191128 | 1.51E-06 | TTC31, CCDC142 |
| rs11517049 | -0.17866 | 0.037051 | 1.42E-06 | HPS1-AS1 |
| rs115186334 | 0.174927 | 0.0381998 | 4.67E-06 | / |
| rs115475653 | -0.26281 | 0.0549287 | 1.71E-06 | / |
| rs115520044 | 0.098504 | 0.0155472 | 2.36E-10 | / |
| rs11568438 | -0.24417 | 0.0531486 | 4.35E-06 | SLC7A7 |
| rs11568591 | 0.148619 | 0.0324267 | 4.58E-06 | ABCC3 |
| rs115713394 | 0.139589 | 0.029064 | 1.56E-06 | / |
| rs11585983 | 0.092196 | 0.0193116 | 1.80E-06 | / |
| rs116004675 | -0.11632 | 0.0250474 | 3.42E-06 | / |
| rs11601631 | -0.1187 | 0.024321 | 1.06E-06 | / |
| rs11612103 | 0.269876 | 0.0518479 | 1.94E-07 | LOC105369715 |
| rs11616521 | -0.0942 | 0.0156171 | 1.62E-09 | LOC124903230 |
| rs11625879 | -0.10936 | 0.0228895 | 1.77E-06 | / |
| rs11626972 | 0.104255 | 0.0193478 | 7.11E-08 | ACOT2, HEATR4 |
| rs11640635 | -0.18618 | 0.0359512 | 2.23E-07 | LOC101927605 |
| rs116476465 | 0.081419 | 0.0169676 | 1.60E-06 | FMNL2 |
| rs116517089 | -0.26087 | 0.0566089 | 4.06E-06 | PLD5 |
| rs116582827 | 0.09665 | 0.0200042 | 1.36E-06 | / |
| rs11661616 | -0.24425 | 0.0246942 | 4.55E-23 | / |
| rs1168041 | -0.13838 | 0.030269 | 4.84E-06 | DOCK7 |
| rs11693959 | -0.07064 | 0.0149328 | 2.24E-06 | GTF3C2 |
| rs11695023 | -0.19765 | 0.0422601 | 2.91E-06 | / |
| rs116972847 | -0.23511 | 0.048356 | 1.16E-06 | PPP1R3B-DT |
| rs1171616 | -0.15972 | 0.0331411 | 1.44E-06 | SLC16A9 |
| rs117178571 | 0.136722 | 0.0299415 | 4.96E-06 | MIR548XHG |
| rs117204314 | -0.25517 | 0.0557388 | 4.69E-06 | / |
| rs117226891 | 0.125198 | 0.0272407 | 4.31E-06 | RPAP3 |
| rs11722698 | -0.1036 | 0.021763 | 1.93E-06 | NDST3, LOC107986307 |
| rs117238367 | 0.130807 | 0.0266699 | 9.36E-07 | / |
| rs117249153 | 0.193768 | 0.0211895 | 5.99E-20 | ANKS1B |
| rs117432550 | 0.4417 | 0.0849249 | 1.98E-07 | ATP8A2 |
| rs117449060 | -0.0783 | 0.0168222 | 3.25E-06 | / |
| rs117579136 | -0.15972 | 0.0337688 | 2.25E-06 | PPP1R12C |
| rs11761910 | -0.09927 | 0.021246 | 2.98E-06 | PDE1C |
| rs11764572 | -0.22198 | 0.0478302 | 3.47E-06 | / |
| rs117846635 | 0.078248 | 0.0165514 | 2.27E-06 | / |
| rs11792609 | -0.10728 | 0.016712 | 1.37E-10 | GNA14 |
| rs117955899 | -0.08762 | 0.0174245 | 4.93E-07 | LDLR |
| rs117958693 | 0.129217 | 0.0236765 | 4.83E-08 | TLE1-DT |
| rs118105919 | -0.12811 | 0.0271744 | 2.43E-06 | / |
| rs118110285 | 0.085836 | 0.0163843 | 1.62E-07 | / |
| rs118143972 | -0.25849 | 0.0560531 | 4.00E-06 | LOC107984887 |
| rs11898850 | 0.081147 | 0.0164143 | 7.67E-07 | SFXN5 |
| rs11930628 | 0.082992 | 0.0165385 | 5.22E-07 | / |
| rs12099786 | -0.12081 | 0.0252936 | 1.79E-06 | GRIP1 |
| rs12129379 | -0.16035 | 0.0326478 | 9.04E-07 | / |
| rs12201824 | -0.16535 | 0.0348722 | 2.12E-06 | ILRUN |
| rs12202350 | 0.214509 | 0.0417205 | 2.72E-07 | / |
| rs12414218 | -0.25775 | 0.0530526 | 1.18E-06 | PAPSS2 |
| rs12437952 | 0.218206 | 0.0448042 | 1.11E-06 | / |
| rs12462427 | 0.186011 | 0.0372574 | 5.96E-07 | ZNF536 |
| rs12481288 | -0.07481 | 0.016048 | 3.13E-06 | PCSK2 |
| rs12505581 | 0.133469 | 0.0267905 | 6.29E-07 | / |
| rs1256522 | -0.07897 | 0.0163742 | 1.42E-06 | / |
| rs12603413 | 0.080272 | 0.017475 | 4.36E-06 | / |
| rs1263613 | 0.080762 | 0.0173576 | 3.27E-06 | KLF7 |
| rs12652257 | 0.320767 | 0.0659023 | 1.13E-06 | MCC |
| rs12659954 | -0.11891 | 0.0243709 | 1.06E-06 | / |
| rs12669002 | -0.20891 | 0.0454962 | 4.40E-06 | / |
| rs12672832 | -0.14504 | 0.0305613 | 2.08E-06 | / |
| rs12695769 | -0.34591 | 0.0731596 | 2.27E-06 | LOC100507389 |
| rs12716675 | 0.229447 | 0.0484519 | 2.18E-06 | LOC105370102 |
| rs12737465 | -0.13554 | 0.0282592 | 1.62E-06 | PBX1, PBX1-AS1 |
| rs12771096 | -0.07254 | 0.0153419 | 2.27E-06 | / |
| rs12798644 | 0.092956 | 0.019685 | 2.33E-06 | SHANK2 |
| rs12878001 | -0.20507 | 0.0387141 | 1.18E-07 | SYNE2 |
| rs12904367 | -0.24452 | 0.0512147 | 1.80E-06 | / |
| rs12917680 | -0.20662 | 0.0446108 | 3.63E-06 | C16orf96 |
| rs12918852 | -0.26785 | 0.0564531 | 2.09E-06 | LOC107984831 |
| rs13081352 | 0.1201 | 0.0162007 | 1.23E-13 | / |
| rs13107084 | -0.13814 | 0.0294771 | 2.78E-06 | / |
| rs13150543 | 0.080039 | 0.0170735 | 2.76E-06 | PPM1K-DT |
| rs13199873 | 0.080814 | 0.0163596 | 7.82E-07 | KLC4 |
| rs13227023 | 0.108494 | 0.0164438 | 4.17E-11 | / |
| rs1323009 | -0.08854 | 0.0169889 | 1.87E-07 | / |
| rs13237261 | 0.233396 | 0.038273 | 1.07E-09 | / |
| rs1324440 | -0.20197 | 0.0394489 | 3.06E-07 | LOC107986638 |
| rs13281473 | 0.357278 | 0.070223 | 3.62E-07 | MSC-AS1 |
| rs1331432 | -0.43144 | 0.0166023 | 6.99E-149 | / |
| rs13319552 | 0.104982 | 0.014827 | 1.44E-12 | / |
| rs13333226 | 0.184693 | 0.0159261 | 4.28E-31 | UMOD |
| rs1333584 | -0.12746 | 0.0249845 | 3.37E-07 | / |
| rs13382655 | -0.34365 | 0.0518914 | 3.53E-11 | THADA |
| rs13390061 | 0.078413 | 0.0171319 | 4.72E-06 | / |
| rs1354034 | 0.233796 | 0.0496776 | 2.52E-06 | ARHGEF3 |
| rs1375284 | 0.199622 | 0.0418304 | 1.82E-06 | NAV3 |
| rs1381273 | 0.07175 | 0.0147395 | 1.13E-06 | / |
| rs138461190 | -0.14823 | 0.0164498 | 2.05E-19 | PECR |
| rs138472585 | 0.095486 | 0.0147244 | 8.88E-11 | / |
| rs13874 | -0.10352 | 0.021216 | 1.06E-06 | SLC25A26 |
| rs138762595 | -0.10219 | 0.0209522 | 1.08E-06 | UGT2B15 |
| rs138800756 | -0.23408 | 0.0336427 | 3.46E-12 | / |
| rs138901164 | 0.075242 | 0.0160512 | 2.76E-06 | RBFOX1 |
| rs139066 | 0.083859 | 0.0177147 | 2.20E-06 | / |
| rs139105296 | -0.20432 | 0.0429754 | 1.99E-06 | IQCM |
| rs139380031 | -0.13917 | 0.0294797 | 2.35E-06 | LOC105375421 |
| rs139448719 | -0.22363 | 0.0469981 | 1.95E-06 | ARMC7 |
| rs139614101 | 0.076298 | 0.0158837 | 1.56E-06 | ARHGAP18 |
| rs139685306 | 0.155627 | 0.0319538 | 1.11E-06 | / |
| rs139721493 | -0.13195 | 0.0284221 | 3.45E-06 | BICC1 |
| rs140460278 | -0.34643 | 0.0680361 | 3.55E-07 | / |
| rs1405100 | 0.303512 | 0.0595407 | 3.44E-07 | SCD5, LOC124900727 |
| rs1408273 | 0.070786 | 0.0153154 | 3.80E-06 | LOC124901285 |
| rs141476650 | 0.131383 | 0.0267793 | 9.29E-07 | LOC100507006 |
| rs141532226 | 0.071702 | 0.015674 | 4.77E-06 | / |
| rs141569914 | -0.11689 | 0.0249222 | 2.73E-06 | GRM7 |
| rs141771065 | -0.17561 | 0.0357674 | 9.12E-07 | LOC105378126 |
| rs141856445 | -0.09849 | 0.0212369 | 3.52E-06 | ATP2C1 |
| rs142298775 | 0.077841 | 0.0165995 | 2.74E-06 | CHST12 |
| rs142426909 | 0.11489 | 0.022903 | 5.27E-07 | / |
| rs142771392 | -0.34007 | 0.0690937 | 8.57E-07 | KLF17 |
| rs143142288 | -0.08923 | 0.0186133 | 1.64E-06 | MATN2 |
| rs143911730 | 0.227419 | 0.0488298 | 3.20E-06 | LINC02220 |
| rs1443156 | 0.094476 | 0.0153515 | 7.55E-10 | / |
| rs1446355 | -0.24896 | 0.0527151 | 2.33E-06 | / |
| rs144858221 | 0.096323 | 0.0209724 | 4.37E-06 | GOT1-DT |
| rs145325641 | -0.17333 | 0.037254 | 3.28E-06 | VEZT |
| rs145778134 | 0.192565 | 0.0421372 | 4.88E-06 | / |
| rs146004765 | 0.07796 | 0.0168535 | 3.73E-06 | / |
| rs146029022 | -0.1727 | 0.034649 | 6.22E-07 | / |
| rs146216027 | 0.098321 | 0.0212006 | 3.52E-06 | TOX |
| rs146330218 | -0.18838 | 0.019895 | 2.84E-21 | FAM189A1 |
| rs146354326 | 0.328224 | 0.0685884 | 1.71E-06 | / |
| rs146629144 | -0.15071 | 0.0321522 | 2.77E-06 | / |
| rs1468626 | -0.0819 | 0.0170952 | 1.66E-06 | / |
| rs1474521 | 0.073242 | 0.0148983 | 8.83E-07 | NCAM2 |
| rs1478210 | 0.082173 | 0.0163295 | 4.85E-07 | UNC13C |
| rs148641172 | 0.097574 | 0.0204297 | 1.79E-06 | PTPN2 |
| rs148685789 | -0.12053 | 0.024091 | 5.65E-07 | ANO3 |
| rs148925249 | 0.08767 | 0.0165347 | 1.14E-07 | CYP7B1 |
| rs149076654 | -0.09542 | 0.0197205 | 1.31E-06 | UBR5 |
| rs149251287 | 0.074987 | 0.016403 | 4.84E-06 | / |
| rs149510848 | 0.086743 | 0.0151612 | 1.06E-08 | GMDS |
| rs1495741 | -0.07295 | 0.0159499 | 4.79E-06 | NAT2 |
| rs1500836 | 0.108892 | 0.0236638 | 4.19E-06 | / |
| rs150162267 | 0.28735 | 0.0474107 | 1.35E-09 | CXCL13 |
| rs150221731 | -0.0881 | 0.0185624 | 2.07E-06 | MOGAT3 |
| rs150287579 | -0.08785 | 0.0179071 | 9.31E-07 | GALNTL6 |
| rs1503281 | -0.12777 | 0.0260927 | 9.75E-07 | CSMD1 |
| rs150600000 | 0.135619 | 0.0222352 | 1.07E-09 | HOMER1 |
| rs151009964 | -0.13115 | 0.0239185 | 4.18E-08 | ALK |
| rs151271070 | 0.081982 | 0.0176418 | 3.37E-06 | CNNM1 |
| rs1532085 | 0.216552 | 0.0470042 | 4.08E-06 | / |
| rs1596732 | -0.39473 | 0.0153632 | 1.40E-145 | / |
| rs1683095 | -0.08766 | 0.0175054 | 5.52E-07 | LOC105370647 |
| rs16848493 | 0.109314 | 0.018413 | 2.91E-09 | LAD1 |
| rs16858780 | 0.073906 | 0.0159953 | 3.83E-06 | CHRD |
| rs16947101 | -0.1404 | 0.0161165 | 2.99E-18 | / |
| rs16986570 | 0.094692 | 0.0204598 | 3.69E-06 | / |
| rs17008088 | -0.19875 | 0.0417302 | 1.91E-06 | FOXP1 |
| rs17013669 | -0.20471 | 0.0414567 | 7.89E-07 | / |
| rs17030870 | 0.096998 | 0.0194661 | 6.26E-07 | THADA |
| rs17042843 | 0.3476 | 0.0748714 | 3.44E-06 | / |
| rs17107008 | 0.269886 | 0.0568336 | 2.05E-06 | / |
| rs17130957 | 0.369815 | 0.0744814 | 6.86E-07 | / |
| rs17239730 | 0.156391 | 0.0331249 | 2.34E-06 | COL14A1 |
| rs17281792 | -0.15791 | 0.0210476 | 6.27E-14 | SMIM21, LOC105372200 |
| rs17324835 | 0.079904 | 0.0162935 | 9.39E-07 | LOC105379013 |
| rs174603 | -0.10231 | 0.022097 | 3.65E-06 | / |
| rs17600560 | -0.10092 | 0.0220975 | 4.95E-06 | PRKG1 |
| rs17684132 | -0.15431 | 0.03307 | 3.07E-06 | ESRRG |
| rs17691714 | 0.186529 | 0.039373 | 2.16E-06 | LGALSL-DT |
| rs17698082 | -0.32885 | 0.06964 | 2.33E-06 | / |
| rs17779334 | 0.110165 | 0.0223981 | 8.72E-07 | ST8SIA4 |
| rs17821152 | -0.13986 | 0.0296992 | 2.49E-06 | SULT1C2 |
| rs17821316 | 0.165801 | 0.0170576 | 2.48E-22 | LIPC |
| rs1784649 | 0.087384 | 0.0183149 | 1.83E-06 | RDX |
| rs181147464 | -0.16128 | 0.0326836 | 8.03E-07 | / |
| rs181311 | -0.20949 | 0.0452835 | 3.72E-06 | / |
| rs181833059 | 0.16997 | 0.0368981 | 4.10E-06 | HMCN1 |
| rs181910869 | -0.24805 | 0.0511614 | 1.25E-06 | PPFIA2 |
| rs182081003 | -0.09537 | 0.0158215 | 1.66E-09 | AKAP6 |
| rs1829257 | -0.12557 | 0.0267413 | 2.66E-06 | LOC105370732, LOC105370733 |
| rs183453 | 0.163133 | 0.0338571 | 1.45E-06 | / |
| rs185067858 | -0.0982 | 0.0214383 | 4.64E-06 | / |
| rs186029333 | -0.07538 | 0.0158605 | 2.01E-06 | / |
| rs186452796 | -0.07559 | 0.0156852 | 1.44E-06 | / |
| rs1871395 | -0.09449 | 0.0201498 | 2.74E-06 | SLCO1B1 |
| rs187513276 | -0.22779 | 0.0490354 | 3.39E-06 | / |
| rs1875205 | 0.111935 | 0.0238475 | 2.68E-06 | GRIN2A |
| rs1876077 | -0.11776 | 0.0172855 | 9.57E-12 | / |
| rs187882577 | -0.08467 | 0.0176503 | 1.61E-06 | LOC100507053 |
| rs188125142 | 0.074258 | 0.0159004 | 3.01E-06 | LOC124901810 |
| rs188133144 | 0.112486 | 0.0160489 | 2.40E-12 | / |
| rs189257248 | -0.09435 | 0.0195543 | 1.40E-06 | DFFB |
| rs189771170 | 0.146166 | 0.0317592 | 4.18E-06 | SLC30A5 |
| rs190712692 | 0.116046 | 0.0251039 | 3.79E-06 | / |
| rs191245370 | 0.095521 | 0.0208001 | 4.38E-06 | / |
| rs191737893 | 0.072042 | 0.0154811 | 3.26E-06 | AHCYL2 |
| rs191815311 | -0.07563 | 0.0160002 | 2.28E-06 | / |
| rs192123848 | 0.089226 | 0.0162296 | 3.85E-08 | SP2-AS1 |
| rs192540541 | 0.172519 | 0.0371775 | 3.48E-06 | KIAA1549L |
| rs194493 | 0.107632 | 0.0174859 | 7.49E-10 | / |
| rs196612 | 0.079756 | 0.0172012 | 3.54E-06 | / |
| rs200257 | -0.24201 | 0.0512745 | 2.36E-06 | / |
| rs2011040 | -0.07575 | 0.0162135 | 2.98E-06 | / |
| rs201330921 | 0.103054 | 0.0177496 | 6.40E-09 | CADM2 |
| rs2016106 | 0.083998 | 0.0164871 | 3.49E-07 | / |
| rs2049544 | -0.07545 | 0.0161752 | 3.09E-06 | / |
| rs2059409 | -0.19491 | 0.0413081 | 2.38E-06 | TNS1 |
| rs2073257 | 0.330598 | 0.0698449 | 2.21E-06 | SEZ6L, SEZ6L-AS1 |
| rs2083631 | 0.130435 | 0.0185658 | 2.13E-12 | LINC02548 |
| rs212100 | -0.19156 | 0.0152189 | 2.48E-36 | LEP1GSC049 |
| rs2140199 | 0.078168 | 0.0168473 | 3.49E-06 | / |
| rs2160387 | 0.166357 | 0.0362649 | 4.49E-06 | SLC1A4 |
| rs2170226 | 0.148079 | 0.0285315 | 2.10E-07 | / |
| rs2189569 | -0.11719 | 0.0248632 | 2.44E-06 | TMEM196, LOC107986774 |
| rs220359 | -0.10603 | 0.022734 | 3.10E-06 | / |
| rs2215327 | -0.07376 | 0.0153798 | 1.62E-06 | CNTNAP2 |
| rs224945 | 0.203401 | 0.0419647 | 1.25E-06 | / |
| rs2251423 | 0.151937 | 0.0325279 | 3.00E-06 | CADPS |
| rs2252533 | 0.07566 | 0.0153457 | 8.21E-07 | / |
| rs2271823 | -0.37158 | 0.0803103 | 3.71E-06 | MELTF, MELTF-AS1 |
| rs2289329 | 0.34391 | 0.0723429 | 2.00E-06 | IVD |
| rs2299639 | 0.079708 | 0.014837 | 7.78E-08 | ABCC8 |
| rs2342209 | -0.26855 | 0.0517048 | 2.06E-07 | / |
| rs235017 | 0.095729 | 0.0201532 | 2.03E-06 | / |
| rs2381400 | 0.15627 | 0.0339312 | 4.11E-06 | RGP1 |
| rs239649 | -0.2356 | 0.0515573 | 4.89E-06 | / |
| rs2407782 | 0.143287 | 0.0298606 | 1.60E-06 | TMEM117 |
| rs2440685 | -0.08167 | 0.0171118 | 1.82E-06 | NR2F2-AS1, LOC112268156 |
| rs2505722 | 0.066738 | 0.0132644 | 4.87E-07 | / |
| rs2518814 | -0.06542 | 0.013457 | 1.17E-06 | DGCR5 |
| rs2526103 | -0.09091 | 0.0171111 | 1.08E-07 | THSD7A |
| rs2534565 | 0.070141 | 0.0149348 | 2.65E-06 | / |
| rs2582788 | 0.121803 | 0.023935 | 3.60E-07 | / |
| rs2585174 | -0.19667 | 0.0417832 | 2.51E-06 | LY6K |
| rs2637258 | -0.4563 | 0.0990409 | 4.08E-06 | LRMDA |
| rs2639989 | 0.200189 | 0.0433222 | 3.82E-06 | PTGR3 |
| rs2640726 | 0.192213 | 0.0418088 | 4.28E-06 | / |
| rs2660831 | 0.214896 | 0.0459496 | 2.91E-06 | / |
| rs266104 | 0.189886 | 0.0398345 | 1.87E-06 | / |
| rs267135 | 0.262591 | 0.0551949 | 1.96E-06 | / |
| rs2721848 | 0.260939 | 0.0391401 | 2.61E-11 | / |
| rs2731726 | 0.09495 | 0.0197856 | 1.60E-06 | SH3PXD2B |
| rs273900 | -0.32014 | 0.0661841 | 1.32E-06 | / |
| rs2744738 | -0.0948 | 0.0206828 | 4.57E-06 | / |
| rs2749530 | -0.09171 | 0.0198934 | 4.02E-06 | / |
| rs2770319 | 0.159849 | 0.0334388 | 1.75E-06 | STPG4 |
| rs2776037 | -0.0795 | 0.017018 | 2.99E-06 | / |
| rs279407 | -0.15749 | 0.0313648 | 5.13E-07 | / |
| rs281377 | 0.093888 | 0.0201705 | 3.24E-06 | / |
| rs2815415 | 0.10712 | 0.0186147 | 8.69E-09 | CCDC18 |
| rs281584 | 0.266849 | 0.056696 | 2.52E-06 | / |
| rs28380236 | -0.22905 | 0.0493444 | 3.45E-06 | / |
| rs28415045 | -0.06933 | 0.0148416 | 2.99E-06 | / |
| rs28415528 | 0.076074 | 0.014461 | 1.44E-07 | IGF1R |
| rs28422045 | -0.13885 | 0.0299465 | 3.54E-06 | / |
| rs28460841 | 0.147175 | 0.0320129 | 4.28E-06 | / |
| rs28505302 | 0.079314 | 0.0161787 | 9.47E-07 | SULT2A1 |
| rs28510524 | -0.1015 | 0.0206289 | 8.65E-07 | / |
| rs2861789 | -0.19327 | 0.0408229 | 2.20E-06 | CTNNA2 |
| rs28655196 | 0.067649 | 0.013888 | 1.11E-06 | / |
| rs28661681 | -0.11091 | 0.023109 | 1.59E-06 | LOC105377269 |
| rs2881485 | -0.09154 | 0.0178946 | 3.13E-07 | SYT9 |
| rs289558 | -0.09358 | 0.0182989 | 3.15E-07 | / |
| rs2912547 | -0.10573 | 0.019815 | 9.51E-08 | PCTP |
| rs293435 | 0.150745 | 0.0327822 | 4.26E-06 | / |
| rs2979782 | 0.123971 | 0.0249959 | 7.06E-07 | MTUS1 |
| rs3012140 | 0.111602 | 0.0225337 | 7.32E-07 | / |
| rs3088051 | 0.106652 | 0.0227359 | 2.72E-06 | ULK1 |
| rs3110095 | 0.097849 | 0.0209031 | 2.85E-06 | TMIGD1 |
| rs3121314 | -0.10873 | 0.0228908 | 2.03E-06 | / |
| rs3122550 | -0.10536 | 0.0229109 | 4.25E-06 | / |
| rs34050359 | -0.21266 | 0.0441112 | 1.43E-06 | LINC02436 |
| rs342697 | 0.164477 | 0.0347191 | 2.17E-06 | / |
| rs34301912 | 0.079913 | 0.0170512 | 2.78E-06 | / |
| rs34321271 | 0.10851 | 0.0229104 | 2.18E-06 | / |
| rs34372981 | 0.108047 | 0.0234711 | 4.16E-06 | CTXND2, LOC107985204 |
| rs34413922 | -0.15121 | 0.0327898 | 4.00E-06 | SGF29 |
| rs34437475 | 0.270483 | 0.0573789 | 2.43E-06 | LOC105372760 |
| rs34484301 | 0.313528 | 0.0626295 | 5.55E-07 | LINC01881 |
| rs34488829 | 0.087967 | 0.0192246 | 4.75E-06 | / |
| rs3465 | 0.39072 | 0.0155622 | 4.17E-139 | ACAT2 |
| rs34708625 | -0.0692 | 0.0140159 | 7.94E-07 | / |
| rs34736275 | -0.07268 | 0.0147469 | 8.29E-07 | / |
| rs34841713 | -0.19695 | 0.042509 | 3.60E-06 | / |
| rs34903233 | -0.16147 | 0.0270844 | 2.50E-09 | GALNTL6 |
| rs34941228 | 0.254375 | 0.0556694 | 4.89E-06 | / |
| rs35404636 | -0.06919 | 0.0150823 | 4.49E-06 | TMEM132B |
| rs35520264 | 0.073542 | 0.0141791 | 2.14E-07 | PPARG |
| rs35593326 | -0.09608 | 0.0196532 | 1.02E-06 | / |
| rs35695799 | -0.4745 | 0.0617383 | 1.52E-14 | / |
| rs36004306 | 0.07241 | 0.0152058 | 1.92E-06 | ASPM |
| rs36043527 | -0.27029 | 0.058657 | 4.07E-06 | LOC100506474 |
| rs36113945 | -0.10796 | 0.0171366 | 2.98E-10 | / |
| rs37370 | 0.077811 | 0.0159954 | 1.15E-06 | AGXT2 |
| rs3740065 | 0.081281 | 0.0152811 | 1.04E-07 | ABCC2 |
| rs3746805 | 0.094225 | 0.0184 | 3.04E-07 | / |
| rs3754686 | 0.141592 | 0.0289812 | 1.03E-06 | MCM6 |
| rs3757131 | -0.10584 | 0.0224912 | 2.53E-06 | SLC17A1 |
| rs3762499 | 0.106579 | 0.0228737 | 3.17E-06 | DLX2, DLX2-DT |
| rs377077993 | 0.079699 | 0.0174504 | 4.94E-06 | / |
| rs3803476 | 0.080003 | 0.0167012 | 1.67E-06 | IGF1R |
| rs3811444 | 0.092878 | 0.0200769 | 3.73E-06 | TRIM58 |
| rs3826886 | -0.1383 | 0.0295717 | 2.91E-06 | PPP6R1, TMEM86B |
| rs389269 | -0.08409 | 0.0178391 | 2.43E-06 | / |
| rs3927165 | 0.083363 | 0.0164398 | 3.96E-07 | PVT1 |
| rs3934638 | -0.26315 | 0.0512196 | 2.78E-07 | / |
| rs3944510 | 0.270295 | 0.0587777 | 4.25E-06 | / |
| rs3957147 | 0.366791 | 0.0778402 | 2.45E-06 | / |
| rs40279 | 0.22332 | 0.0472754 | 2.31E-06 | / |
| rs4148324 | -0.09528 | 0.0206039 | 3.76E-06 | UGT1A10, UGT1A8, UGT1A7, UGT1A6, UGT1A5, UGT1A9, UGT1A4, UGT1A1, UGT1A3 |
| rs420430 | -0.07754 | 0.0158421 | 9.86E-07 | / |
| rs4241207 | 0.074554 | 0.0163226 | 4.93E-06 | RFX8 |
| rs4241304 | -0.19438 | 0.0396235 | 9.32E-07 | NDUFA10 |
| rs4257284 | 0.174731 | 0.0366763 | 1.90E-06 | ZNF521, LOC105372031 |
| rs4284207 | 0.097631 | 0.0208578 | 2.86E-06 | / |
| rs4294667 | 0.086289 | 0.0181108 | 1.89E-06 | NALF1 |
| rs4353533 | -0.21571 | 0.0399835 | 6.85E-08 | MARCHF10-DT |
| rs4390625 | -0.20059 | 0.0431541 | 3.35E-06 | CDK12 |
| rs4409767 | -0.25576 | 0.049595 | 2.51E-07 | TACC2 |
| rs4517148 | -0.21702 | 0.0441181 | 8.70E-07 | / |
| rs4533344 | 0.08746 | 0.0178912 | 1.02E-06 | / |
| rs454363 | 0.094472 | 0.0199097 | 2.08E-06 | / |
| rs4554032 | -0.16567 | 0.0360073 | 4.20E-06 | / |
| rs45597035 | -0.17476 | 0.0363431 | 1.52E-06 | KLF5 |
| rs459553 | -0.07295 | 0.015816 | 3.99E-06 | / |
| rs4627308 | -0.11461 | 0.0224756 | 3.41E-07 | ATP10A |
| rs4654595 | -0.16423 | 0.0356824 | 4.17E-06 | AJAP1 |
| rs4664114 | -0.109 | 0.0238305 | 4.79E-06 | FMNL2 |
| rs4685976 | 0.254636 | 0.0523993 | 1.18E-06 | LOC105376938, LOC105376939 |
| rs4688978 | -0.22465 | 0.0474177 | 2.16E-06 | JAKMIP1-DT |
| rs4713731 | 0.099567 | 0.0216731 | 4.35E-06 | / |
| rs4721923 | -0.08138 | 0.0168162 | 1.30E-06 | / |
| rs4738772 | -0.11228 | 0.0228602 | 9.03E-07 | / |
| rs4738877 | -0.22486 | 0.0491594 | 4.78E-06 | CLVS1 |
| rs475225 | 0.512248 | 0.10295 | 6.50E-07 | / |
| rs4785580 | 0.099277 | 0.0209969 | 2.27E-06 | DPEP1 |
| rs4790506 | 0.212153 | 0.0446523 | 2.02E-06 | SPATA22, TRPV3 |
| rs479229 | 0.143002 | 0.0311759 | 4.50E-06 |  |
| rs4795346 | -0.1918 | 0.0396529 | 1.32E-06 | PLXDC1, RDM1P5 |
| rs4804669 | -0.14407 | 0.0294983 | 1.04E-06 | ZNF799 |
| rs4806498 | 0.101803 | 0.0206761 | 8.49E-07 | TMC4 |
| rs4810397 | -0.20411 | 0.0425869 | 1.64E-06 | / |
| rs4814176 | -0.17752 | 0.036631 | 1.26E-06 | / |
| rs4822809 | -0.2621 | 0.0546006 | 1.58E-06 | LOC110091768 |
| rs483082 | -0.0745 | 0.0161452 | 3.94E-06 | / |
| rs4852102 | -0.1293 | 0.0279496 | 3.73E-06 | / |
| rs4910140 | -0.23203 | 0.0456552 | 3.73E-07 | AMPD3 |
| rs4917820 | 0.106995 | 0.0217894 | 9.09E-07 | / |
| rs4950876 | -0.08045 | 0.0173692 | 3.62E-06 | PPFIA4 |
| rs495360 | -0.28881 | 0.0626082 | 3.97E-06 | / |
| rs5001310 | 0.091312 | 0.019884 | 4.39E-06 | / |
| rs55942846 | -0.14107 | 0.0300731 | 2.72E-06 | / |
| rs55958922 | 0.143028 | 0.0302635 | 2.29E-06 | ANKRD28 |
| rs55971546 | 0.163969 | 0.0340016 | 1.42E-06 | SLC10A2 |
| rs55986203 | 0.091258 | 0.0197201 | 3.70E-06 | / |
| rs56109834 | 0.317693 | 0.0634921 | 5.62E-07 | / |
| rs56113850 | -0.27899 | 0.0422415 | 3.98E-11 | CYP2A6 |
| rs56137600 | -0.07361 | 0.0157238 | 2.85E-06 | / |
| rs56165099 | 0.208733 | 0.0146443 | 4.26E-46 | SLCO1B1 |
| rs56311231 | 0.181231 | 0.0323216 | 2.06E-08 | ZFHX3 |
| rs56335545 | -0.17386 | 0.0278879 | 4.55E-10 | ATP8B3 |
| rs56387403 | 0.309172 | 0.0675655 | 4.74E-06 | LINC02758 |
| rs56850653 | 0.213538 | 0.0465431 | 4.48E-06 | LOC107987085 |
| rs56996827 | -0.07688 | 0.0165808 | 3.54E-06 | / |
| rs5751247 | 0.155339 | 0.0338956 | 4.59E-06 | TCF20 |
| rs57743625 | 0.163792 | 0.0352811 | 3.44E-06 | SLCO1B1 |
| rs58423714 | -0.22502 | 0.0336174 | 2.18E-11 | LAMC1 |
| rs59066200 | 0.32928 | 0.0709887 | 3.51E-06 | LOC105369165 |
| rs59162951 | 0.334245 | 0.0719446 | 3.39E-06 | ABLIM3 |
| rs591858 | 0.227302 | 0.0488471 | 3.27E-06 | / |
| rs595653 | -0.08575 | 0.0178665 | 1.59E-06 | / |
| rs59761505 | 0.215555 | 0.0416656 | 2.30E-07 | CSMD1 |
| rs5997787 | 0.102738 | 0.0224877 | 4.91E-06 | OSBP2 |
| rs60253740 | 0.093648 | 0.0199474 | 2.67E-06 | LCT |
| rs6034162 | 0.506448 | 0.100887 | 5.17E-07 | MACROD2 |
| rs6040971 | 0.134498 | 0.0198794 | 1.33E-11 | BTBD3 |
| rs60556060 | 0.375997 | 0.0722127 | 1.92E-07 | LOC105371357 |
| rs606241 | 0.094453 | 0.0204049 | 3.68E-06 | / |
| rs6066667 | -0.13352 | 0.0283537 | 2.49E-06 | LOC105372641 |
| rs6081085 | 0.154576 | 0.028915 | 9.00E-08 | LOC124904876 |
| rs6103589 | -0.10257 | 0.0224338 | 4.83E-06 | TOX2 |
| rs6108309 | 0.100086 | 0.0198351 | 4.51E-07 | / |
| rs6109233 | 0.295721 | 0.0646458 | 4.77E-06 | / |
| rs6111089 | -0.16111 | 0.0335691 | 1.59E-06 | KIF16B |
| rs6116934 | 0.078918 | 0.0171569 | 4.23E-06 | SHLD1 |
| rs6123665 | 0.104822 | 0.0228315 | 4.41E-06 | / |
| rs61352371 | 0.229656 | 0.0489658 | 2.73E-06 | / |
| rs61517008 | -0.31827 | 0.0656665 | 1.26E-06 | / |
| rs61760359 | 0.158617 | 0.031854 | 6.38E-07 | PRKCA |
| rs61762011 | 0.075144 | 0.0162274 | 3.65E-06 | PIK3C2A |
| rs61833951 | 0.071103 | 0.015402 | 3.90E-06 | SDCCAG8 |
| rs61880478 | 0.074905 | 0.0162915 | 4.27E-06 | / |
| rs61883501 | -0.07507 | 0.015701 | 1.74E-06 | LINC02545 |
| rs61895821 | -0.10698 | 0.016367 | 6.31E-11 | LOC105369325 |
| rs61897793 | 0.071783 | 0.0154758 | 3.51E-06 | FADS2 |
| rs61929004 | 0.395909 | 0.0755289 | 1.59E-07 | / |
| rs61987308 | 0.090937 | 0.0196759 | 3.81E-06 | LOC124903315 |
| rs62130083 | -0.11348 | 0.0170855 | 3.10E-11 | / |
| rs62132803 | 0.075315 | 0.0160173 | 2.58E-06 | / |
| rs62206283 | 0.083713 | 0.0169143 | 7.45E-07 | DUSP15 |
| rs62245941 | -0.15387 | 0.0334966 | 4.36E-06 | TBC1D5 |
| rs62248512 | -0.08344 | 0.01729 | 1.39E-06 | / |
| rs62287171 | -0.08405 | 0.0168631 | 6.21E-07 | ATP13A4 |
| rs62296161 | -0.20048 | 0.0417543 | 1.58E-06 | / |
| rs62354638 | 0.079102 | 0.0165155 | 1.67E-06 | SLC1A3 |
| rs62383014 | -0.34615 | 0.0736682 | 2.62E-06 | / |
| rs62411867 | -0.12012 | 0.0174777 | 6.29E-12 | COL21A1 |
| rs62418960 | 0.114136 | 0.0203386 | 2.00E-08 | TRDN, LOC105377984 |
| rs62467675 | -0.07984 | 0.0165876 | 1.48E-06 | ASB4 |
| rs62471956 | 0.217813 | 0.044631 | 1.06E-06 | / |
| rs62471957 | 0.094645 | 0.0194679 | 1.16E-06 | CYP3A43 |
| rs62493774 | 0.163364 | 0.0331891 | 8.56E-07 | LOC100128993 |
| rs62513764 | 0.265506 | 0.0567604 | 2.90E-06 | CPA6, ARFGEF1-DT |
| rs62525488 | 0.090061 | 0.0195155 | 3.93E-06 | LOC105369147 |
| rs648658 | 0.181455 | 0.0384871 | 2.42E-06 | / |
| rs6506246 | 0.223799 | 0.0449504 | 6.40E-07 | / |
| rs6517376 | -0.22026 | 0.0160746 | 9.85E-43 | SIM2, LOC107985492 |
| rs6564915 | -0.08012 | 0.0169971 | 2.43E-06 | / |
| rs6572232 | 0.070026 | 0.0153092 | 4.78E-06 | / |
| rs6580847 | 0.172818 | 0.0346782 | 6.24E-07 | / |
| rs66482844 | 0.193644 | 0.0408225 | 2.10E-06 | / |
| rs6682605 | -0.07896 | 0.0170476 | 3.62E-06 | LOC124904421 |
| rs6687264 | -0.15634 | 0.0276376 | 1.54E-08 | / |
| rs67165923 | -0.09796 | 0.0212249 | 3.93E-06 | / |
| rs6737049 | -0.08316 | 0.0172016 | 1.34E-06 | FBXO41 |
| rs6749227 | 0.078433 | 0.016625 | 2.38E-06 | STRN |
| rs6755215 | 0.182594 | 0.0376269 | 1.22E-06 | RAB17-DT |
| rs6759896 | 0.077705 | 0.0165062 | 2.51E-06 | / |
| rs676206 | -0.08275 | 0.0166773 | 6.99E-07 | / |
| rs67816826 | 0.165078 | 0.0355318 | 3.39E-06 | / |
| rs6781900 | 0.160689 | 0.0351545 | 4.86E-06 | / |
| rs6792151 | 0.164317 | 0.0347905 | 2.32E-06 | PLD1 |
| rs6836669 | -0.09542 | 0.0200515 | 1.95E-06 | CNGA1 |
| rs6839814 | -0.1016 | 0.0220793 | 4.19E-06 | PABPC4L |
| rs6842362 | -0.35801 | 0.0730174 | 9.44E-07 | / |
| rs6854192 | -0.18044 | 0.0371053 | 1.16E-06 | GALNTL6 |
| rs686587 | -0.10379 | 0.0225904 | 4.34E-06 | / |
| rs687059 | 0.234398 | 0.0491926 | 1.89E-06 | / |
| rs6963576 | 0.175216 | 0.0348896 | 5.11E-07 | SUGCT, LOC105375242 |
| rs6980809 | 0.286138 | 0.061695 | 3.52E-06 | TRPA1 |
| rs7010461 | -0.10247 | 0.02177 | 2.52E-06 | IDO1 |
| rs710362 | 0.331516 | 0.0670856 | 7.75E-07 | / |
| rs71316533 | -0.25178 | 0.0551418 | 4.97E-06 | / |
| rs7136437 | -0.07397 | 0.0158354 | 2.99E-06 | / |
| rs71576259 | 0.307868 | 0.0540215 | 1.21E-08 | / |
| rs7201200 | 0.22906 | 0.0475688 | 1.47E-06 | / |
| rs7206111 | -0.12727 | 0.0152285 | 6.40E-17 | PRDM7 |
| rs7234333 | 0.325052 | 0.0677247 | 1.59E-06 | DSG4, DSG1-AS1 |
| rs7241978 | -0.17536 | 0.017849 | 8.82E-23 | DLGAP1, DLGAP1-AS4 |
| rs72629155 | 0.07811 | 0.0166655 | 2.77E-06 | SMIM17, ZNF71-SMIM17 |
| rs72650372 | 0.219426 | 0.0466383 | 2.54E-06 | RP1 |
| rs72663498 | -0.22881 | 0.0211352 | 2.59E-27 | / |
| rs72664200 | 0.0964 | 0.0184563 | 1.76E-07 | SSBP3 |
| rs72700111 | -0.12799 | 0.0275805 | 3.48E-06 | LINC01681 |
| rs7270451 | -0.25417 | 0.0545729 | 3.20E-06 | DOK5 |
| rs72727918 | 0.134689 | 0.020857 | 1.06E-10 | / |
| rs72754978 | 0.074932 | 0.0163227 | 4.42E-06 | SLC28A1 |
| rs72761651 | -0.136 | 0.0245522 | 3.04E-08 | TARS3 |
| rs72808733 | -0.22869 | 0.0471773 | 1.25E-06 | PRKCE |
| rs72842528 | -0.08875 | 0.0152991 | 6.58E-09 | CRTAC1 |
| rs72864158 | -0.09745 | 0.0198269 | 8.88E-07 | / |
| rs72916458 | 0.597461 | 0.0149484 | 1.00E-200 | / |
| rs72958660 | -0.08452 | 0.0175047 | 1.37E-06 | / |
| rs72977885 | -0.0774 | 0.0161861 | 1.74E-06 | / |
| rs72987261 | -0.34692 | 0.0429563 | 6.69E-16 | / |
| rs73146320 | 0.414681 | 0.038367 | 3.15E-27 | / |
| rs73178107 | 0.286252 | 0.0606341 | 2.35E-06 | / |
| rs73179138 | 0.172275 | 0.0360487 | 1.76E-06 | / |
| rs73179662 | 0.266372 | 0.0537622 | 7.25E-07 | CSMD1 |
| rs7320739 | -0.39457 | 0.0615163 | 1.42E-10 | LOC107984626 |
| rs73232175 | -0.0716 | 0.0155915 | 4.38E-06 | / |
| rs73233394 | 0.665751 | 0.0144899 | 1.00E-200 | PKNOX1 |
| rs73234134 | -0.10126 | 0.0202127 | 5.45E-07 | LOC105374510 |
| rs73234777 | -0.10512 | 0.0227125 | 3.69E-06 | PCBP3 |
| rs7324476 | -0.19506 | 0.0406884 | 1.63E-06 | / |
| rs73250695 | 0.392011 | 0.0845833 | 3.58E-06 | LGI2, LOC124900684 |
| rs732912 | 0.314823 | 0.058801 | 8.60E-08 | / |
| rs73371153 | -0.27051 | 0.0552449 | 9.75E-07 | MYOM1 |
| rs73423516 | 0.079312 | 0.0170032 | 3.09E-06 | CCDC178 |
| rs73486205 | 0.1005 | 0.017266 | 5.86E-09 | GALR1, LOC124904329 |
| rs73516870 | -0.22444 | 0.0478698 | 2.75E-06 | MGRN1 |
| rs736418 | 0.332543 | 0.0702254 | 2.19E-06 | / |
| rs736780 | -0.25843 | 0.0562104 | 4.27E-06 | / |
| rs73746675 | 0.134812 | 0.0285376 | 2.31E-06 | PACSIN1 |
| rs73855271 | 0.108988 | 0.0214208 | 3.62E-07 | GTF2E1 |
| rs73963343 | -0.35692 | 0.0771389 | 3.71E-06 | / |
| rs7398567 | -0.11504 | 0.0231905 | 7.03E-07 | CERS5, LOC124902931 |
| rs74377562 | 0.267843 | 0.0555883 | 1.45E-06 | / |
| rs74388472 | -0.13285 | 0.0257605 | 2.51E-07 | CABLES1 |
| rs74461768 | -0.09903 | 0.0216306 | 4.69E-06 | / |
| rs746872 | 0.128184 | 0.0250385 | 3.06E-07 | / |
| rs74709126 | -0.1637 | 0.0337602 | 1.24E-06 | NKAIN2 |
| rs74947424 | 0.399949 | 0.0861825 | 3.47E-06 | / |
| rs74962281 | -0.14398 | 0.0303609 | 2.11E-06 | / |
| rs74964662 | -0.3293 | 0.0672747 | 9.83E-07 | / |
| rs74985032 | -0.14099 | 0.0272605 | 2.32E-07 | PLCG2 |
| rs75008620 | 0.141631 | 0.0289023 | 9.57E-07 | INPP5D |
| rs75017029 | -0.09676 | 0.0211347 | 4.69E-06 | / |
| rs75039646 | 0.144064 | 0.0283602 | 3.78E-07 | / |
| rs75063835 | 0.378886 | 0.0828719 | 4.83E-06 | MIR320B1 |
| rs75134544 | 0.320444 | 0.0688219 | 3.22E-06 | ESR1 |
| rs75142236 | -0.10256 | 0.0222443 | 4.01E-06 | ALK |
| rs7514506 | 0.298436 | 0.0619834 | 1.47E-06 | BRINP2 |
| rs75148797 | 0.093084 | 0.0201146 | 3.70E-06 | CTNND2 |
| rs75191864 | -0.09237 | 0.0191655 | 1.44E-06 | SEMA6A, SEMA6A-AS1 |
| rs75311326 | -0.06858 | 0.0150155 | 4.94E-06 | CRYBG1 |
| rs7577494 | 0.277191 | 0.0588257 | 2.45E-06 | LINC01122 |
| rs7582470 | 0.153897 | 0.0325357 | 2.24E-06 | GLI2 |
| rs76003961 | -0.08693 | 0.018616 | 3.02E-06 | CTNNA2 |
| rs7620877 | 0.071396 | 0.0146021 | 1.01E-06 | / |
| rs7636303 | -0.09047 | 0.0178105 | 3.78E-07 | CLSTN2 |
| rs76420143 | -0.4869 | 0.0637928 | 2.30E-14 | / |
| rs7651439 | 0.221377 | 0.0473213 | 2.89E-06 | MB21D2 |
| rs7654754 | 0.165722 | 0.0326878 | 3.98E-07 | SHROOM3 |
| rs76577804 | 0.095535 | 0.0208206 | 4.47E-06 | PAAF1 |
| rs76636050 | -0.17516 | 0.0364118 | 1.51E-06 | / |
| rs76637894 | -0.39203 | 0.0848273 | 3.81E-06 | / |
| rs76652113 | 0.308775 | 0.0640808 | 1.45E-06 | / |
| rs76763222 | 0.165583 | 0.0345252 | 1.62E-06 | / |
| rs76793825 | -0.1359 | 0.0284968 | 1.85E-06 | / |
| rs76891935 | 0.141607 | 0.0306594 | 3.86E-06 | LOC105370324 |
| rs76978746 | -0.1254 | 0.0234542 | 8.96E-08 | / |
| rs77075152 | -0.11104 | 0.0202168 | 3.96E-08 | / |
| rs7707728 | -0.20677 | 0.0448397 | 4.00E-06 | / |
| rs77104570 | -0.24939 | 0.0423424 | 3.86E-09 | FUT9, LOC105377905 |
| rs7718900 | -0.1439 | 0.0302932 | 2.03E-06 | SLC6A19, SLC6A18 |
| rs77487713 | -0.09327 | 0.0201818 | 3.81E-06 | / |
| rs77519877 | 0.090778 | 0.0189375 | 1.64E-06 | / |
| rs7757300 | 0.099228 | 0.0214475 | 3.72E-06 | / |
| rs7758685 | -0.16931 | 0.0317919 | 1.01E-07 | POLR1C, LOC105375070 |
| rs77627840 | -0.08534 | 0.0167397 | 3.43E-07 | / |
| rs7780066 | 0.086538 | 0.0182529 | 2.13E-06 | AKR1D1 |
| rs7782140 | 0.120774 | 0.0244714 | 8.00E-07 | VPS41 |
| rs77924615 | 0.100974 | 0.0212596 | 2.04E-06 | PDILT |
| rs77944838 | -0.27719 | 0.0576317 | 1.51E-06 | LINC01619 |
| rs7826908 | -0.1653 | 0.0346912 | 1.89E-06 | PLEKHA2 |
| rs78334557 | 0.091082 | 0.0178504 | 3.35E-07 | ROBO1 |
| rs78429682 | -0.13087 | 0.0280433 | 3.06E-06 | / |
| rs78525462 | -0.2378 | 0.0504053 | 2.38E-06 | PDGFC |
| rs78602320 | 0.30314 | 0.0657018 | 3.95E-06 | PRKCB, MIR1273H |
| rs78708293 | -0.32368 | 0.0683386 | 2.18E-06 | SORCS2 |
| rs7874009 | 0.170195 | 0.0366796 | 3.48E-06 | DENND1A, MIR7150 |
| rs78755500 | -0.3292 | 0.0718593 | 4.63E-06 | LINC00670 |
| rs78788136 | 0.215867 | 0.0455421 | 2.14E-06 | / |
| rs79121515 | -0.08406 | 0.0183615 | 4.69E-06 | CNTNAP4 |
| rs79237419 | -0.16152 | 0.0339812 | 2.00E-06 | ZBTB20 |
| rs79253762 | -0.20316 | 0.0228284 | 5.62E-19 | DCTD |
| rs79318581 | -0.36358 | 0.0391833 | 1.71E-20 | PKNOX2 |
| rs79361541 | 0.164794 | 0.0346627 | 1.99E-06 | SMCHD1 |
| rs79374656 | -0.18578 | 0.0222872 | 7.70E-17 | MTHFD1 |
| rs7948250 | -0.16593 | 0.0350737 | 2.24E-06 | GRM5 |
| rs79497827 | -0.24004 | 0.0515319 | 3.19E-06 | LINC00448 |
| rs79529411 | -0.19276 | 0.0417518 | 3.90E-06 | / |
| rs79549670 | 0.14731 | 0.0288093 | 3.17E-07 | / |
| rs79591343 | -0.14835 | 0.0312395 | 2.05E-06 | / |
| rs7964924 | -0.07923 | 0.0171471 | 3.82E-06 | ITPR2 |
| rs79656653 | 0.08265 | 0.0174858 | 2.28E-06 | ERICH6 |
| rs79799178 | -0.26329 | 0.0543279 | 1.26E-06 | ALMS1 |
| rs79829592 | -0.31265 | 0.0677642 | 3.96E-06 | / |
| rs79976360 | -0.08425 | 0.0182641 | 3.98E-06 | TENM2 |
| rs80039270 | -0.23363 | 0.0169838 | 4.70E-43 | / |
| rs80051868 | 0.098152 | 0.0208509 | 2.51E-06 | / |
| rs80175591 | -0.23947 | 0.0479539 | 5.92E-07 | / |
| rs8017983 | -0.15856 | 0.0330685 | 1.63E-06 | / |
| rs80254170 | -0.12777 | 0.0262416 | 1.12E-06 | IGF2R, CHP1P2 |
| rs8032949 | 0.281404 | 0.0615001 | 4.75E-06 | / |
| rs80332390 | 0.125017 | 0.0270508 | 3.81E-06 | / |
| rs8050812 | -0.07772 | 0.0167539 | 3.50E-06 | MYL11 |
| rs8523 | 0.079509 | 0.0170098 | 2.95E-06 | ELOVL2 |
| rs876481 | 0.099747 | 0.0203452 | 9.45E-07 | / |
| rs887829 | 0.304425 | 0.0649121 | 2.73E-06 | UGT1A1 |
| rs916688 | 0.157637 | 0.0314609 | 5.43E-07 | / |
| rs919217 | 0.250002 | 0.0512622 | 1.08E-06 | / |
| rs923251 | -0.08011 | 0.0151952 | 1.35E-07 | / |
| rs9323181 | -0.07533 | 0.0155685 | 1.31E-06 | LINC01599 |
| rs933271 | 0.261711 | 0.0563354 | 3.39E-06 | / |
| rs9345201 | 0.083774 | 0.0175914 | 1.91E-06 | / |
| rs9370390 | 0.106106 | 0.021834 | 1.18E-06 | HCRTR2 |
| rs9405331 | -0.28611 | 0.0608013 | 2.53E-06 | RREB1 |
| rs9409510 | 0.094801 | 0.0200904 | 2.37E-06 | ZNF169 |
| rs943385 | -0.07665 | 0.0153477 | 5.90E-07 | / |
| rs943402 | 0.081371 | 0.0174785 | 3.23E-06 | / |
| rs943485 | 0.105679 | 0.0216767 | 1.09E-06 | / |
| rs9459497 | -0.17303 | 0.0373774 | 3.67E-06 | PDE10A |
| rs9473939 | 0.444139 | 0.0962703 | 3.96E-06 | / |
| rs9497988 | 0.127081 | 0.0275962 | 4.12E-06 | SASH1 |
| rs9514591 | -0.102 | 0.0211662 | 1.44E-06 | LOC124903248 |
| rs9536317 | 0.20248 | 0.0443384 | 4.95E-06 | / |
| rs9558610 | -0.25046 | 0.0521205 | 1.55E-06 | LINC00343 |
| rs9594738 | 0.104272 | 0.0220598 | 2.28E-06 | TNFSF11 |
| rs9611880 | -0.21488 | 0.046019 | 3.02E-06 | / |
| rs970488 | -0.1665 | 0.0350304 | 2.01E-06 | / |
| rs9749077 | 0.073319 | 0.0160384 | 4.84E-06 | RELB |
| rs9803636 | 0.301739 | 0.0643829 | 2.78E-06 | SLC44A3-AS1 |
| rs9816900 | 0.240137 | 0.0258275 | 1.44E-20 | / |
| rs9818127 | 0.111129 | 0.0237909 | 3.00E-06 | PAQR9-AS1 |
| rs9822689 | 0.256754 | 0.0434236 | 3.36E-09 | / |
| rs9838187 | -0.18521 | 0.040367 | 4.47E-06 | GRM7 |
| rs9847968 | -0.1928 | 0.040967 | 2.52E-06 | / |
| rs99780 | -0.20874 | 0.043882 | 1.97E-06 | FADS2 |
